# Engineered CRISPR/Cas9 enzymes improve discrimination by slowing DNA cleavage to allow release of off-target DNA

**DOI:** 10.1038/s41467-020-17411-1

**Published:** 2020-07-17

**Authors:** Mu-Sen Liu, Shanzhong Gong, Helen-Hong Yu, Kyungseok Jung, Kenneth A. Johnson, David W. Taylor

**Affiliations:** 1 Department of Molecular Biosciences, Austin, TX 78712 USA; 2 Institute for Cellular and Molecular Biology, Austin, TX 78712 USA; 3 Center for Systems and Synthetic Biology, University of Texas at Austin, Austin, TX 78712 USA; 4 LIVESTRONG Cancer Institutes, Dell Medical School, Austin, TX 78712 USA

**Keywords:** Enzyme mechanisms, CRISPR-Cas9 genome editing

## Abstract

CRISPR/Cas9 is a programmable genome editing tool widely used for biological applications and engineered Cas9s have increased discrimination against off-target cleavage compared with wild-type Streptococcus pyogenes (SpCas9) in vivo. To understand the basis for improved discrimination against off-target DNA containing important mismatches at the distal end of the guide RNA, we performed kinetic analyses on the high-fidelity (Cas9-HF1) and hyper-accurate (HypaCas9) engineered Cas9 variants. We show that DNA cleavage is impaired by more than 100- fold for the high-fidelity variants. The high-fidelity variants improve discrimination by slowing the observed rate of cleavage without increasing the rate of DNA rewinding and release. The kinetic partitioning favors release rather than cleavage of a bound off-target substrate only because the cleavage rate is so low. Further improvement in discrimination may require engineering increased rates of dissociation of off-target DNA.

## Abstract (web summary)

Engineered high-fidelity Cas9s have increased discrimination against off-targets. Kinetic analyses of Cas9-HF1 and HypaCas9 engineered Cas9 variants show that their DNA cleavage is impaired by more than 100- fold, which leads to release rather than cleavage of a bound off-target substrate.

## Introduction

CRISPR/Cas9 has become widely used for precise genome editing applications in basic research and represents a promising tool for future applications^1–6^. However, Cas9 endonucleases show unintended off-target cleavage events that pose serious limitations to Cas9-based gene therapies. Several Cas9 enzyme variants have been developed that lead to significant improvements in discrimination in vivo, including: high-fidelity (Cas9-HF1: N467A, R661A, Q695A, and Q926A mutations)^7^, enhanced specificity (eSpCas9(1.1): K848A, K1003A, and R1060A mutations)^8^ and hyper-accurate (HypaCas9: N692A, M694A, Q695A, and H698A mutations)^9^. However, substrate discrimination is a kinetic phenomenon, and the basis for target discrimination and the guidelines for further improving fidelity remain unclear^10–14^. Recently, we showed that DNA cleavage is fast and DNA unwinding constitutes the rate-limiting and specificity-determining step for on-target cleavage by SpCas9^15^. Later single molecule studies confirmed that DNA unwinding appeared to be the rate-limiting step for SpCas9 but further suggested that DNA unwinding is the determinant for specificity with both wild-type and high-fidelity Cas9 variants, Cas9-HF1 and eCas9(1.1), for 3 bp PAM-distal mismatches^16^. However, single molecule kinetic studies that lack a parallel measurement of the rates of DNA cleavage provide limited information to establish the mechanistic basis for DNA cleavage specificity, which is a function of all steps leading up to the first largely irreversible reaction^17^.

**Fig. 1:**
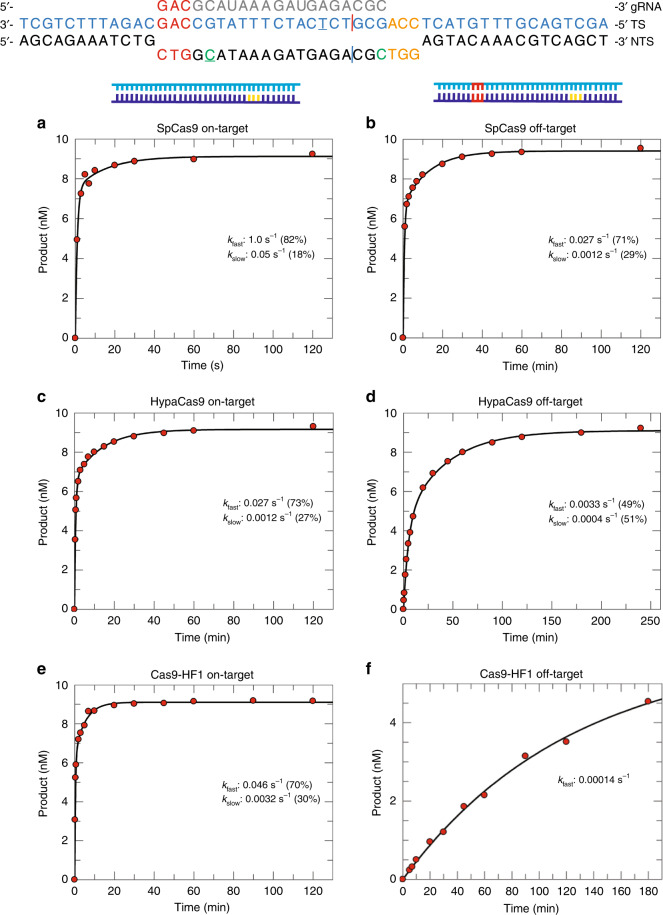
PAM-distal mismatches dramatically slow cleavage by HypaCas9 and Cas9-HF1. Schematic of target DNA substrate. PAM site is orange; target sequence is blue; mismatches in the PAM distal region are red; gRNA is colored gray; cleavage sites are shown as red and blue vertical lines for the HNH and RuvC domains, respectively; tC^o^ labeled site at position −16 nt or −1 nt in the non-target strand is green. Cy3 and Cy5 labeled sites at position −6 nt and −16 nt in the target strand and non-target strand, respectively, are underlined. A time course of cleavage of on-target DNA (10 nM) was monitored with 28 nM of each enzyme. Data were fit to a double-exponential equation and the percentage of the total signal occurring with each decay rate is indicated. SpCas9 cleavage of on-target (**a**) (from Gong et al.^15^) and off-target (**b**) DNA. **c, d** HypaCas9 cleavage with on-target (**c**) and off-target (**d**) DNA. **e, f** Cas9-HF1 cleavage of on-target (**e**) and off-target (**f**) DNA. Data in **f** were fit to a single-exponential function.

Because the PAM-distal mismatches (Fig. 1) have been shown to play an important role in selectivity as observed in single molecule experiments of both DNA unwinding and conformational dynamics within the enzyme^18–22^, we chose to continue our study of the effects of this off-target activity using single turnover kinetic analysis. These studies include parallel measurements of conformational changes and DNA cleavage and are interpreted globally to yield a single unifying model defining intrinsic rate constants governing specificity. We show that high-fidelity variants increase discrimination largely through kinetic partitioning, favoring release rather than cleavage of off-target substrates.

## Results

### HypaCas9 and Cas9-HF1 show slow observed rates of cleavage

We begin our kinetic analyses by measuring the enzyme active site concentration for each of the Cas9 variants^23–25^. Measuring the amount of product formed in a titration of enzyme with increasing concentrations of DNA revealed active site concentrations of 31 nM, 26 nM, 23 nM for SpCas9, HypaCas9, and Cas9-HF1, respectively, for enzyme samples with a 100 nM nominal concentration based on absorbance at 280 nm (Supplementary Fig. 1a–d). We also measured the active site concentrations of SpCas9 and HiFiCas9^25^ from Integrated DNA Technologies (IDT) and observed similar concentrations of active enzyme (Supplementary Fig. 1e–f). It is important to note that, in each case, the concentration of DNA required to saturate the signal was equal to the concentration of product formed, which eliminates concerns that some of the enzyme might bind DNA but not react. All subsequent experiments were set up using the concentration of active enzyme determined in the active site titration.

To compare the kinetics of on- or off-target DNA substrates of SpCas9 with the engineered variants, we first examined the time course of target strand (HNH) cleavage for each enzyme (Fig. 1 and Supplementary Fig. 2). Data were fit with either a single- or double-exponential function using Eq. (1) or Eq. (2), respectively.

1 $$Y = A_1e^{ - \lambda _1t} + C$$

where *Y* represents concentration of cleavage product, *A*_*1*_ represents the amplitude, and ***λ***_1_ represents the observed decay rate (eigenvalue)^17^.

2 $$Y = A_1e^{ - \lambda _1t} + A_2e^{ - \lambda _2t} + C$$

where *Y* represents concentration of cleavage product, *A*_*1*_ represents the amplitude and ***λ***_1_ represents the observed rate for the first phase. *A*_*2*_ represents the amplitude and ***λ***_2_ represents the observed rate for the second phase.

Comparison of the observed cleavage decay rates of on- and off-target substrates by SpCas9 shows that the 3 bp PAM-distal mismatch slows the enzyme 13-fold (from 1 s^−1^ to 0.076 s^−1^). Both high-fidelity Cas9 variants dramatically decrease the observed decay rate for cleavage of on-target DNA substrates 21- to 35-fold compared to SpCas9 (0.028 s^−1^ for HypaCas9 and 0.047 s^−1^ for Cas9-HF1 vs 1 s^−1^ for SpCas9). Moreover, HypaCas9 and Cas9-HF1 further reduce the decay rates of off-target DNA cleavage 8- to 290-fold (rates of 0.0033 s^−1^ and 0.00016 s^−1^, respectively) relative to their respective rates with on-target DNA. These data demonstrate dramatic changes in the observed rates of cleavage by the engineered variants with on- and off-target DNA. However, these measurements alone do not define changes in specificity, which is a function of the kinetic partitioning of DNA cleavage versus dissociation, encompassing all steps leading up to the first irreversible step in the pathway^17^, including reversible DNA binding, R-loop formation, HNH domain docking, and DNA cleavage.

### DNA unwinding is largely unchanged with the Cas9 variants

**Fig. 2:**
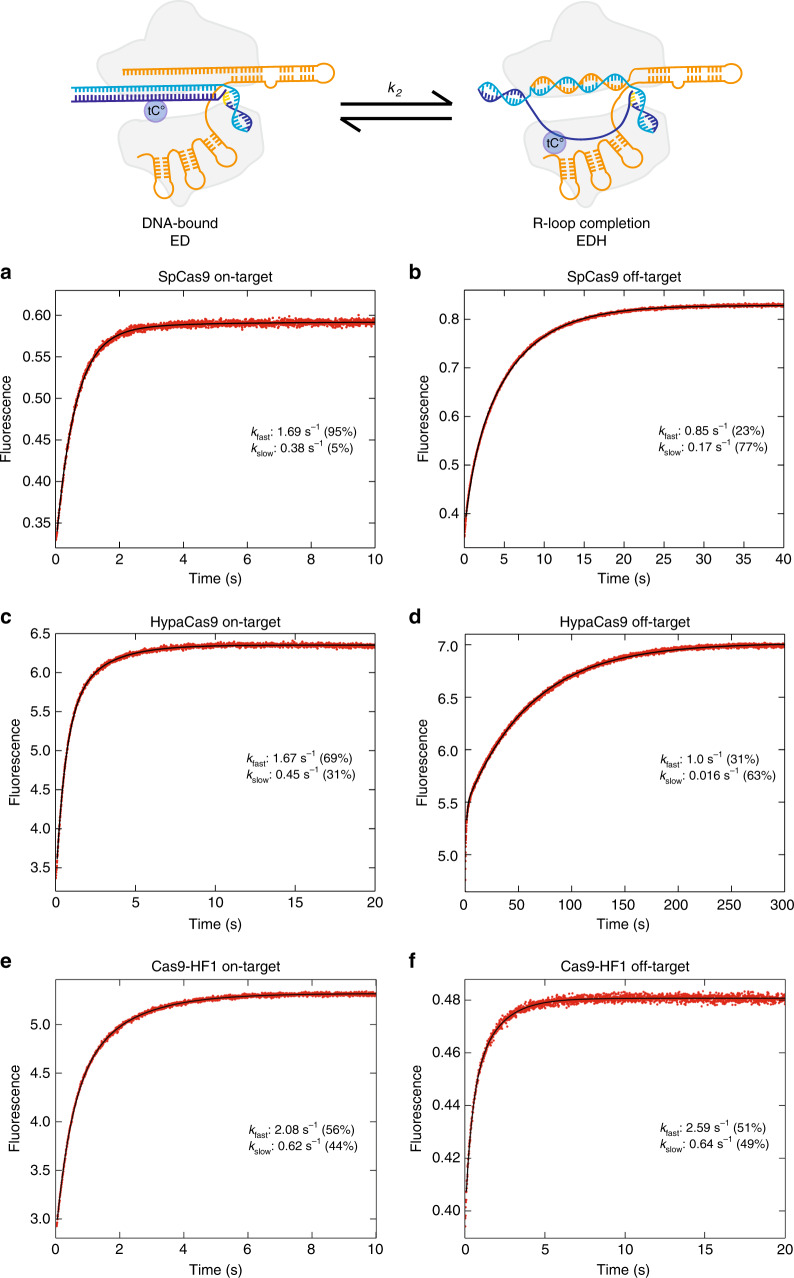
DNA unwinding rates are nearly identical for on-targets and off-targets. R-loop formation decay rates were measured by the monitoring the fluorescence increase from tC^o^ (position −16 in the non-target strand) as a function of time after mixing enzyme (28 nM) with DNA (10 nM) in the presence of 10 mM Mg^2+^. Data were fit to a double exponential function to get the decay rates shown. **a, b** SpCas9 (28 nm) with on-target (**a**) and off-target (**b**) DNA, **c, d** HypaCas9 with on-target (**c**) and off-target (**d**) DNA. **e, f** Cas9-HF1 with on-target (**e**) and off-target (**f**) DNA.

Since our previous work identified R-loop formation as rate-limiting for on-target cleavage and others subsequently suggested that R-loop formation and rewinding rates may dictate enzyme specificity for SpCas9 and Cas9-HF1^16^, we tested whether HypaCas9 would display similar kinetics. To directly measure the rates of R-loop formation for all enzymes, we used a stopped-flow assay based on measuring fluorescence of tC^o^ at −16 nt, a fluorescent tricyclic cytosine analog that is quenched by base stacking in dsDNA so that opening of the duplex results in a large increase in fluorescence. Our control experiments using both our tC^o^- and 2-AP-labeled base analog substrates at positions −16, −9, and −1 nt, respectively (Supplementary Fig. 3), show that neither of the analogs affect the observed decay rate for DNA cleavage. The chemical structures of tC^o^ and 2AP are much less bulky than larger Cy3 and Cy5 labels and less likely to interfere with enzyme kinetics (Supplementary Fig. 4). In the presence of Mg^2+^, SpCas9, HypaCas9, and Cas9-HF1 unwind the on-target DNA substrate with nearly identical decay rates (~2 s^−1^) (Fig. 2). Surprisingly, the decay rate of R-loop formation for off-target DNA substrates for all Cas9 variants was also largely unchanged (between 0.85 s^−1^ and 2.59 s^−1^). Therefore, DNA unwinding is not rate-limiting and is not correlated with observed rates of cleavage for the high-fidelity variants, unlike SpCas9.

**Fig. 3:**
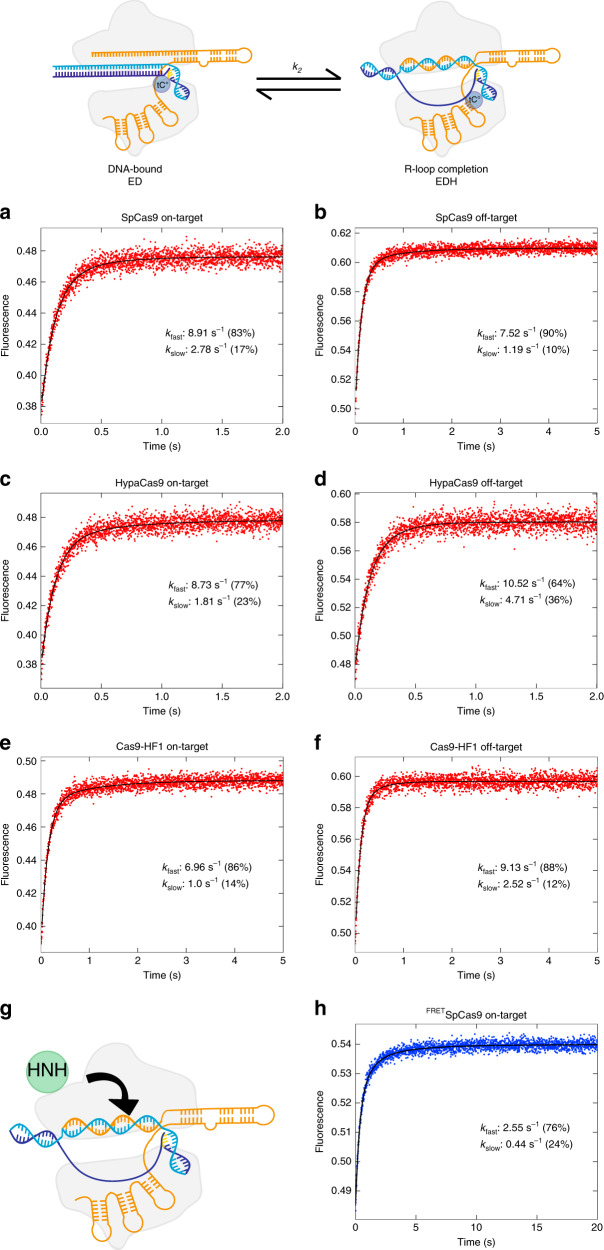
DNA unwinding occurs more rapidly near the PAM. R-loop formation decay rates were measured by the monitoring the fluorescence increase from tC^o^ (position −1 in the non-target strand) as a function of time after mixing enzyme (28 nM) with DNA (10 nM) in the presence of 10 mM Mg^2+^. Data were fit to a double exponential function to get the decay rates shown. **a, b** SpCas9 (28 nm) with on-target (**a**) and off-target (**b**) DNA, **c, d** HypaCas9 with on-target (**c**) and off-target (**d**) DNA. **e, f** Cas9-HF1 with on-target (**e**) and off-target (**f**) DNA. **g** Cartoon of the HNH domain rearrangement. **h** The ^FRET^SpCas9 labeled with Cy3 and Cy5 pair at C867 and C355, respectively, was used to measure the HNH domain motion during cleavage of on-target substrates.

Given the location of the tC^o^ at −16 nt, it is likely that our measurements reflected a step late in the unwinding process. For comparison, we measured the decay rate for R-loop formation using tC^o^ labeled at a position immediately proximal to the PAM (position −1 nt). After rapidly mixing Cas9-RNA with the on-target DNA substrate, we observed a marked increase in fluorescence as the DNA unwinds the tC^o^ base analog. We fit the data to a double-exponential to determine the major decay rate of 10 s^−1^ (Fig. 3a–f). Intriguingly, these results indicate that once a PAM site is engaged with the enzyme, initial unwinding of the DNA is faster than observed at −16 nt. These data suggest that the net observed decay rate of unwinding observed at −16 nt may be a function of several fast unwinding step leading to complete unwinding. However, because no lag was observed in the kinetics, it appears that the faster, earlier partial unwinding steps lead to a final rate-limiting step of full unwinding, measured at the −16 nt position. Although further studies are needed to examine the effects of mismatches at earlier stages of unwinding, our current measurement provides the best estimate of the net rate of R-loop formation. The decay rate measured for R-loop formation using this label is indistinguishable for both SpCas9 and the high-fidelity variants on all substrates tested, so the rates for DNA unwinding appear to be unchanged by the engineered Cas9 enzymes.

To correlate the rates of R-loop formation with steps involved in HNH-domain docking onto the target strand, we measured the enzyme conformational change using stopped-flow analysis on a Cas9 which was labeled with Cy3 and Cy5 as described previously^18^. Briefly, a Cysteine-light version of Cas9 was labeled with Cy3 at amino acid 355 and Cy5 at amino acid 867. The FRET efficiency increases when the HNH domain rearranges to the catalytically active state (Fig. 3g). After rapidly mixing the FRET-pair-labeled Cas9 with a perfectly matched on-target DNA, we observed an increase in FRET efficiency, indicating that the HNH domain was rearranging to a catalytically active state. We measured the decay rate for HNH docking to be ~2.5 s^−1^ (Fig. 3h), which is similar to single molecule FRET measurements. Surprisingly, the observed rates of R-loop formation (1.5 s^−1^) and HNH domain docking are very similar, indicating that these steps may be kinetically linked. The slightly faster observed decay rate for HNH domain movement could be due to the reverse reaction as the domain movement comes to equilibrium after or coincident with R-loop formation.

The decay rates of DNA unwinding have also been measured using single molecule methods using a FRET-pair labeled DNA, Cy3 and Cy5 were labeled on position −6 nt of the target strand and -16 nt on the non-target strand, respectively^16^. Therefore, we also tested the FRET-paired DNA substrates in an attempt to correlate the FRET signal with the observed rates of DNA cleavage. First, we tested the substrate previously used in single molecule studies without the Cy3/Cy5 labels^16^, which has a two-nucleotide difference with our substrate, specifically, a T substitution at positions −16 and −18. The decay rate of target strand (HNH) cleavage is similar to that of our substrate (0.7 s^−1^ vs 1 s^−1^, Supplementary Fig. 5a), so sequence context at this position does not have a significant affect. We also tested cleavage with Cy3/Cy5 labeled DNA with this other sequence, and it showed a similar decay rate as our sequence (0.05 s^−1^ vs 0.06 s^−1^, Supplementary Figs. 5b and 6a). Later, we examined the time course of target strand (HNH) cleavage of the Cy3/Cy5 labeled DNA with our sequence for each enzyme (Supplementary Fig. 6). With SpCas9, the reaction of the Cy3/Cy5 labeled DNA follows a single exponential with a markedly reduced decay rate (0.06 s^−1^ vs 1 s^−1^) showing a ~17-fold decrease in the observed decay rate for DNA cleavage compared to unlabeled substrate. The engineered HypaCas9 and Cas9-HF1 also exhibited decreased DNA cleavage rates of 0.0046 s^−1^ and 0.0016 s^−1^, respectively, which are 5.9-fold and 28.75-fold slower than unlabeled substrates measured under identical conditions. It is clear that interference by the Cy3/Cy5 labels alters the effect of the high-fidelity variants on the DNA cleavage rates. Taken together, labeling of the DNA with bulky Cy3 and Cy5 labels dramatically impacted the enzyme. For these reasons, no further studies were performed using the FRET-pair labeled DNA.

### Cas9 specificity is governed by kinetic partitioning

Since enzyme specificity is a function of all steps leading up to the first largely irreversible step, all events prior to DNA cleavage must be considered^17^. To measure the intrinsic cleavage rate, we bypassed the normally rate-limiting R-loop formation step by preincubating SpCas9 with off-target DNA in the absence of Mg^2+^ to allow binding and conformational changes to come to equilibrium to form an SpCas9.DNA complex. We then initiated the chemical reaction by the addition of 10 mM Mg^2+^. In our previous studies, R-loop formation was rate-limiting with SpCas9 reacting with on-target DNA, and the intrinsic cleavage decay rate was faster when measured using the preincubation protocol. Here, with off-target DNA, the rates of HNH and RuvC cleavage were measured to be 0.12 s^−1^ and 0.14 s^−1^, respectively (Supplementary Fig. 7c, d, and Supplementary Fig. 8), which are much slower than the rates of R-loop formation. Intriguingly, these results show that the rate-limiting step in the enzyme pathway of SpCas9 with an off-target is DNA cleavage since the decay rate for R-loop formation we measured was 0.85 s^−1^ (Fig. 2b). These data indicate that discrimination is based, at least in part, on a change in the identity of the rate-limiting step in comparing on- and off-target DNA.

We repeated these experiments with HypaCas9 and Cas9-HF1 with on- or off-target DNA. These results define observed rates for HNH cleavage of 0.035 s^−1^ and 0.0023 s^−1^ for HypaCas9 with on- and off-target substrates, respectively (Supplementary Fig. 7g, k). Observed cleavage rates of Cas9-HF1 for on- and off-target substrates were measured as 0.038 s^−1^ and 0.00014 s^−1^, respectively (Supplementary Fig. 7o, q). These observed cleavage rates are somewhat faster than those measured with the simultaneous addition of DNA and Mg^2+^, indicating that some step other than R-loop formation but preceding DNA cleavage may slow the observed decay rate. Nonetheless, these results show that the observed cleavage rates for on-target DNA are reduced ~100-fold for both HypaCas9 and Cas9-HF1 relative to SpCas9. For off-target DNA, the observed cleavage rates are reduced 50- or 860-fold for HypaCas9 or Cas9-HF1, respectively, relative to SpCas9.

**Fig. 4:**
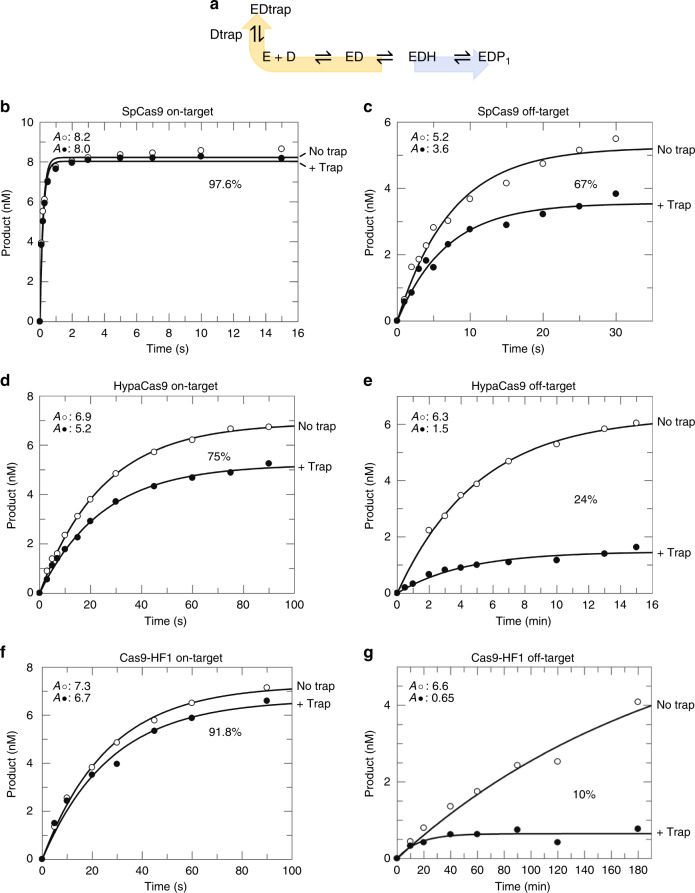
Engineered Cas9s improve specificity through a decreased rate of DNA cleavage. **a** Schematic of DNA trap experiment. **b, c** SpCas9 cleavage of on-target (from Gong et al.^15^) (**b**) or off-target (**c**) DNA. **d, e** HypaCas9 cleavage of on-target (**d**) or off-target (**e**) DNA. **f**, **g** Cas9-HF1 cleavage of on-target (**f**) or off-target (**g**) DNA. Enzyme (28 nM) and labeled DNA (10 nM) were incubated in the absence of Mg^2+^ and the reaction was initiated by adding Mg^2+^ in the presence or absence of an excess of unlabeled DNA trap. *A*_•_ and *A*_◦_ represent the amplitude with (filled circles) and without trap (open circles), respectively. The percentage indicates the fraction of pre-bound DNA committed to going forward for cleavage relative to the reaction in the absence of trap.

Discrimination is not defined solely by the relative rates of DNA cleavage. Rather, because R-loop formation is fast, discrimination is a function of the kinetic partitioning between the rates of DNA release versus cleavage^17,26,27^. In order to quantify the kinetic partitioning, we incubated enzyme and labeled DNA in the absence of Mg^2+^, which allows for R-loop formation to come to equilibrium, but prevents catalysis^15^. We then added Mg^2+^ to initiate the reaction in the presence of a large excess of identical, unlabeled on-target DNA to serve as a trap. Comparison between parallel experiments performed in the presence and absence of the DNA trap provides an estimate for the fractional kinetic partitioning for dissociation versus cleavage of bound DNA (Fig. 4a). Once SpCas9 was bound to on-target DNA, it was cleaved rapidly, and the DNA trap had little effect (Fig. 4b). In contrast, 33% of the off-target DNA disassociated from the enzyme, while ~67% of the DNA was committed to cleavage (Fig. 4c and Supplementary Fig. 9a). These results show that SpCas9 discriminates against the PAM-distal mismatched DNA by decreasing the rate of cleavage, which increases the fraction of DNA that is released rather than cleaved. However, the effect is small so the dissociation rate appears to be slower than cleavage.

Next, we examined the kinetic partitioning for HypaCas9 and Cas9-HF1 bound to on-target DNA (Fig. 4d, f, Supplementary Fig. 9b, d). Our results with HypaCas9 and Cas9-HF1 show that ~75% and ~92% of the on-target DNA was cleaved in the presence of the trap, respectively. The percentage cleaved is smaller than for wild-type SpCas9 because the observed intrinsic cleavage decay rate for on-target DNA by HypaCas9 (0.035 s^−1^) and Cas9-HF1 (0.038 s^−1^) was 100-fold slower than with SpCas9 (4.3 s^−1^). This slower cleavage rate gives time for a small fraction (8 to 25%) of the on-target DNA to dissociate before it is cleaved.

Kinetic partitioning to favor dissociation was enhanced when HypaCas9 and Cas9-HF1 react with off-target DNA because the cleavage rates were further reduced to 0.0023 s^−1^ and 0.00014 s^−1^, respectively (Fig. 4e, g, Supplementary Fig. 9c, e). These rates are 50- to 860-fold slower, respectively, compared to SpCas9 on an off-target substrate. Accordingly, only ~24% and ~10% of the bound off-target DNA was committed to going forward for cleavage by HypaCas9 and Cas9-HF1, respectively, in the presence of trap DNA. Taken together, these results show that the engineered high-fidelity variants acquired improved specificity against the PAM-distal mismatched DNA through a markedly decreased rate of cleavage, which alters kinetic partitioning to favor release rather than cleavage of the bound substrate for both on- and off-target DNA, but the effect is larger for off-target DNA. Calculation of the apparent dissociation rate constants using Eq. (3) shows that the high-fidelity variants do not increase the apparent rate of DNA release (Supplementary Table 1).

3 $${\mathrm{Cleavage}}\,{\mathrm{probability}} = \frac{{k_{chem}}}{{k_{off} + k_{chem}}}$$

Rather, the increased discrimination is entirely attributed to decreases in the apparent rate constant for cleavage.

**Fig. 5:**
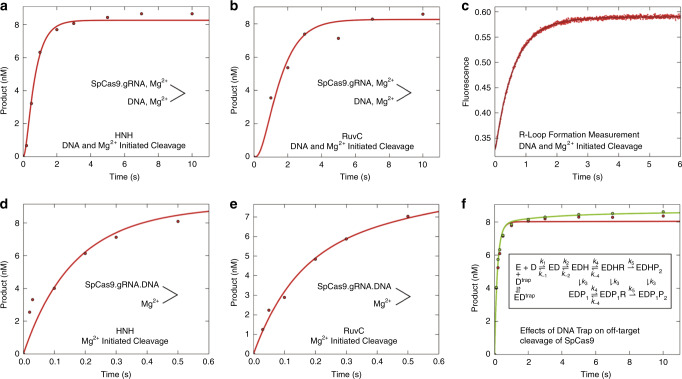
Global fitting of experiments performed to interrogate on-target activity of SpCas9. **a** DNA and Mg^2+^ initiated cleavage by the HNH domain. **b** DNA and Mg^2+^ initiated cleavage by the RuvC domain. **c** R-loop formation rate. **d** Mg^2+^ initiated cleavage by the HNH domain. **e** Mg^2+^ initiated cleavage by the RuvC domain. **f** Effect of DNA trap on kinetic partitioning of Cas9 cleavage. All curves were calculated based on the global fit to the data according to the kinetic model (inset), with rate constants shown in Table 1. Error estimates were based on confidence contour analysis as shown in Fig. 6. E is Cas9.gRNA, D is target DNA, ED is Cas9.gRNA.DNA, EDH is R-loop formation with docking of target strand to HNH, EDHR and EDP_1_R are R-loop formation with docking of non-target strand to RuvC, EDHP_2_ is RuvC cleavage of non-target strand, EDP_1_ is HNH cleavage of the target-strand, EDP_1_P_2_ is cleavage of both strands, D^trap^ is excess unlabeled, perfect-matched DNA. ED^trap^ is Cas9.gRNA.DNA^trap^.

**Fig. 6:**
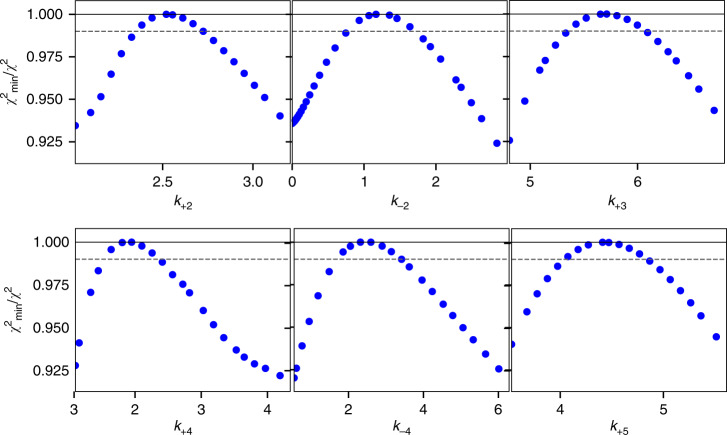
Confidence contour analysis for global data fitting. Confidence contours are shown for fitting data in Fig. 5. Numbered rate constants are defined in our kinetic model (Fig. 5f, inset). The *χ*^2^ threshold (dashed line) was set to 0.99 to define upper and lower limits for each rate constant as described^17,32^. Because the contours were symmetrical with parameters well constrained, we report ± error limits in Table 1. For this analysis a *χ*^2^ threshold was calculated using an F-distribution and was used to define the lower and upper limits for each parameter.

In our descriptions of the kinetics of Cas9 enzymes thus far, the observed decay rates of reactions were estimated based on fitting data to exponential functions, which yield eigenvalues that are usually complex functions of multiple rate constants^17^. To fully understand the kinetics and mechanism, all of the experiments for each enzyme and DNA substrate were fit globally based on numerical integration of the rate equations using a single unified model (Fig. 5 and Supplementary Figs. 10–14). Global data fitting shows that our minimal model (Fig. 5f, inset) accounts for our data and each of the rate constants is well defined based on confidence contour analysis testing the extent to which each parameter is constrained by the data (Fig. 6)^17,28^. Note in particular that our model accounts for the biphasic traces seen in some experiments without having to invoke heterogeneity, and it provides intrinsic rate constants for each step in the pathway including R-loop formation and HNH and RuvC cleavage events. Although it is likely that additional, unresolved structural rearrangements may be required for alignment of catalytic residues for DNA cleavage, these have not yet been resolved as kinetically distinct events. The current model represents a minimal pathway necessary and sufficient to account for all available data, and can be easily expanded as new information becomes available to define additional steps in the pathway.

To understand enzyme specificity, rate constants must be interpreted in the context of all kinetically relevant steps as illustrated in the free energy profile (calculated using Eq. (4) from rate constants in Table 1).

4 $$\Delta {\mathrm{G}}^\ddagger = RT\left( {{\mathrm{ln}}\left( {{\mathrm{A}} \ast kT/h} \right)-{\mathrm{ln}}\left( {k_{obs}} \right)} \right)\,{\mathrm{kcal}}/{\mathrm{mol}}$$

The transmission coefficient *A* = 0.001

**Table 1:** Kinetic parameters for Cas9 enzymes.

	DNA binding and R-loop formation				HNH cleavage	RuvC alignment		RuvC cleavage
Enzyme	1/*K*_1_^1^ (nM)	*K*_d,net_^2^ (nM)	*k*_2_ (s^−1^)	*k*_*-*2_ (s^−1^)	*k*_3_ (s^−1^)	*k*_4_ (s^−1^)	*k*_*-*4_ (s^−1^)	*k*_5_ (s^−1^)
SpCas9 on-target	[3]^3^	[1]^3^	2.5 ± 0.2	1.2 ± 0.4	5.6 ± 0.4	1.8 ± 0.3	2.3 ± 0.6	4.4 ± 0.4
SpCas9 off-target	24	8.1	1.1 ± 0.1	0.5 ± 0.1	0.17 ± 0.01	0.7 ± 0.1	0.30 ± 0.05	0.38 ± 0.02
Hypa on-target	4.3	1.6	0.7 ± 0.03	0.4 ± 0.02	0.042 ± 0.0007	1.0 ± 0.02	0.08 ± 0.006	0.054 ± 0.001
Hypa off-target	52	17	0.8 ± 0.1	0.4 ± 0.01	0.005 ± 0.001	0.04 ± 0.001	0.0004 ± 0.0003	0.008 ± 0.003
Cas9HF1 on-target	1.6	0.12	2.2 ± 0.2	0.2 ± 0.08	0.038 ± 0.002	0.71 ± 0.04	0.02 ± 0.01	0.032 ± 0.002
Cas9HF1 off-target	33	9.8	2.5 ± 2	0.7 ± 0.3	0.0002 ± 0.00002	1.1 ± 0.8	0.4 ± 0.2	0.0002 ± 0.00005

^1^We used a nominal value of *k*_*1*_ = 1 nM^-1^s^-1^ and fit data to derive *k*_*-1*_ to compute *K*_*1*_ = *k*_*-1*_*k*_*1*_.

^2^*K*_*d,net*_ = 1/(*K*_*1*_(1 + *K*_*2*_)), where *K*_2_ = *k*_*2*_/*k*_*-2*_

^3^Values for DNA dissociation rates are a function of *k*_*-1*_ and *k*_*-2*_, and for SpCas9 on-target DNA these values are not well defined by the data because of the low kinetic partitioning for DNA dissociation (Fig. 4a). For global data fitting, we locked rate constants *k*_*-1*_ = 3 s^−1^ at a nominal upper limit. Because this rate constant was not well-defined by the data, locking it at a reasonable upper limit had little effect on the values for other rate constants.

**Fig. 7:**
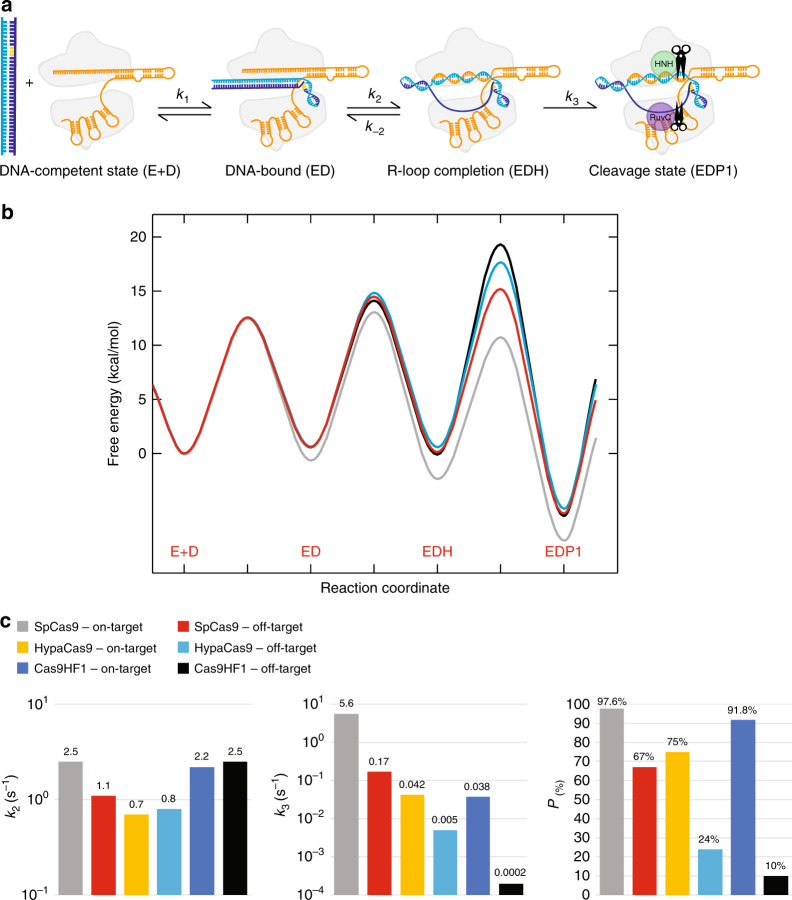
Specificity is governed by the kinetic partitioning of Cas9. **a** Cartoon representation of Cas9 enzymes reaction pathway. **b** Free energy profiles for SpCas9 cleavage of an on-target (gray line) from Gong et al.^15^ and off-target (red line), respectively; HypaCas9 cleavage of an off-target (blue line); and Cas9-HF1 cleavage of an off-target (black line). Each profile was calculated using transition state theory using rate and equilibrium constants that were derived from globally fitting each set of experiments (Table 1). Note the higher barriers for DNA cleavage relative to the lower barrier for the preceding reverse reaction increase the probability of DNA release. **c** Summary bar graph for *k*_2_, *k*_3_, and kinetic partitioning (P).

Because global data fitting provides the rate constants for each relevant step (Figs. 5 and 6), we can construct a *bona fide* free energy profile (Fig. 7b, and Supplementary Figs. 10–14). The free energy profiles comparing SpCas9, HypaCas9, and Cas9-HF1 show a change in rate-limiting and specificity-determining steps. Enzyme specificity is defined by *k*_*cat*_/*K*_m_ and is given by the highest overall barrier relative to the starting state, while the maximum rate, *k*_*cat*_, is defined by the highest local barrier relative to the preceding state, attenuated by any preceding rapid-equilibrium steps. Because the rate constants for R-loop formation do not change significantly with different substrates and enzyme variants, specificity is governed largely by the kinetic partitioning of the Cas9 R-loop, EDH state in our kinetic model (Fig. 5f, inset), to either go forward resulting in irreversible cleavage versus release via re-annealing of the DNA and ejection from the enzyme. The higher overall barriers for cleavage seen with the high-fidelity variants increase the kinetic partitioning probability to favor dissociation of the DNA (Fig. 6c).

## Discussion

Our results are important to rationalize in relation to single molecule studies. Previous studies have measured the rate of transition of the HNH domain from the inactive to the active state on an on-target using single-molecule FRET to be 1.5 s^−1^^20^. This is consistent with our measurements of R-loop formation and HNH domain docking (1.5–2.5 s^−1^), suggesting that HNH docking is limited by the rate-limiting step of R-loop formation. Since SpCas9 has an intrinsic cleavage rate of 4 s^−1^, our calculations demonstrate kinetic partitioning to favor cleavage rather than DNA release so that that R-loop formation and coincident HNH docking constitute the specificity-determining steps for wild-type enzyme with on-target DNA.

Interestingly, the previously determined decay rate for the HNH domain transition for the off-target DNA is 0.05 s^−1^ ^20^. This is consistent with our measurements of the rate of cleavage of an off-target DNA when Mg^2+^ and DNA are simultaneously mixed with SpCas9 of 0.076 s^−1^. Our measurement of the intrinsic cleavage decay rate for SpCas9 on an off-target (0.12 s^−1^) suggests that there may be another conformational change or structural rearrangement that occurs before cleavage, as this transition is not rate-limiting. This also suggests that our measurements using pre-equilibration of Cas9 and DNA in the absence of Mg^2+^ has no effect on the ability of the HNH domain to sample the active state as this intrinsic cleavage rate is faster than that measured with simultaneous addition of Mg^2+^ and DNA.

Previous studies have also measured the decay rate for the conversion of the HNH domain of Cas9-HF1 from the inactive to active state (0.2 s^−1^)^9^. Our data show that the decay rates for cleavage with either simultaneous addition of DNA and Mg^2+^ or pre-equilibration followed by initiation with Mg^2+^ are 0.037 s^−1^. This would again suggest that while HNH domain movement is slower than for SpCas9, it is faster than the observed rate of DNA cleavage and not rate-limiting.

In this study, we provide a more complete understanding of enhanced specificity of high-fidelity Cas9 variants. HypaCas9 and Cas9-HF1 are seriously impaired in terms of enzyme efficiency of on-target DNA cleavage. For each variant, the cleavage rate was 100-fold slower for on-target cleavage as compared to SpCas9. HypaCas9 and Cas9-HF1 gain discrimination mainly through further slowing of the rate of DNA cleavage with off-target DNA, which shifts kinetic partitioning to favor release rather than cleavage of the bound DNA^26,27^. We propose that Cas9 uses an induced-fit mechanism analogous to DNA polymerases, where a conformational change after initial substrate binding (HNH domain movement) is an important determinant of enzyme specificity. For SpCas9, the conformational change is rate-limiting and determines specificity because R-loop formation is largely irreversible and is followed by fast DNA cleavage^15^. The higher-fidelity variants have extraordinarily slow rates of DNA cleavage, allowing for release of the substrate from the enzyme before the irreversible cleavage reaction even though the dissociation rate is slow. Although the high-fidelity variants have increased fidelity, calculated from the *k*_*cat*_*/K*_*m*_ values for on- versus off-target DNA, they achieved this through markedly reduced enzyme efficiency (*k*_*cat*_*/K*_*m*_ for on-target DNA). The slower rates of DNA cleavage may be the result of a slower rate of HNH domain movement, an unfavorable equilibrium for the conformational change, and/or a suboptimal alignment of catalytic residues for DNA cleavage. DNA polymerases have evolved much higher fidelity by dramatically increasing the rate of dissociation of mismatched nucleotides in addition to decreasing the rate of catalysis^26,27^. Improvements in Cas9 specificity without sacrificing efficiency may require engineering an enzyme that leads to more rapid release of off-target DNA.

Engineered Cas9 enzymes achieve improved discrimination against mismatches at the distal end of the guide sequence by decreasing the rate of catalysis. Our results are consistent with the model that the REC3 domain senses whether or not the final 3-nt of the target strand are base-paired with the guide RNA, and that this signal is communicated to the tethered REC2 domain. In this model, the REC2 domain is evicted from its normal position, which allows the HNH domain to dock on the target strand for cleavage^9^. This is likely due to REC3 domain sensing the shape of the substrate, and we hypothesize that duplex formation between the gRNA and target strand is the trigger for the catalytical rearrangement^29,30^. This moderate improvement in discrimination may be sufficient in the context of the full recognition sequence to disfavor off-target cleavage in vivo. However, further improvements to enable gene therapy may require engineered enzymes that increase rates of off-target DNA dissociation without requiring such drastic reductions in efficiency of on-target cleavage.

## Methods

### Expression and purification of Cas9s

Plasmid pMJ806 containing *Streptococcus pyogenes* Cas9 and plasmid pSHS306 containing the HNH FRET variant of *Streptococcus pyogenes* Cas9 were obtained from Addgene (Cambridge, MA). Plasmid pHypaCas9 was a gift from Dr. Ilya Finkelstein at the University of Texas at Austin. For bacterial expression of the recombinant SpCas9 and HypaCas9, plasmids were transformed into BL21-Rosetta 2 (DE3)-competent cells (Millipore). The *E. coli* cells were cultured at 37 °C in LB media (containing working concentration 50 μg/mL kanamycin for SpCas9 and HypaCas9 and 100 μg/mL ampicillin for ^FRET^SpCas9) until the OD_600_ reached to 0.5–0.8, and then Cas9 expression was induced by the addition of 0.5 mM isopropyl-β-D-1-thiogalactopyranoside (IPTG) for 20 h at 18 °C. The His_6_-MBP tagged Cas9 were purified by a combination of affinity, cation exchange, and size exclusion chromatographic steps, essentially as described previously^15^ with following modifications. Briefly, bacterial cells were lysed by sonication in buffer containing 50 mM Tris-HCl, pH 7.5, 10% glycerol, 500 mM NaCl, 2 mM phenylmethylsulfonyl fluoride (PMSF), 0.5 mM TCEP and Pearce protease inhibitor cocktails (Thermo Scientific). Clarified lysate was applied to HisPur ^TM^ Ni-NTA resin (Thermo Scientific) and the resin was washed extensively with buffer containing 50 mM Tris-HCl, pH 7.5, 10% glycerol, 500 mM NaCl, 1 mM PMSF, 0.5 mM TCEP, and 25 mM Imidazole. The bound protein was eluted in 50 mM Tris-HCl, pH 7.5, 10% glycerol, 500 mM NaCl, 1 mM PMSF, 0.5 mM TCEP, and 200 mM Imidazole. The His_6_-MBP-Cas9 fusion protein was cleaved to remove the His_6_-MBP tag by adding the TEV protease to the dialysis tubing (Spectrum labs) during dialysis against buffer containing 20 mM HEPES pH 7.5, 5% glycerol, 150 mM KCl, 2 mM PMSF, and 0.5 mM TCEP. After further purification by SP cation exchange chromatography, the cleaved Cas9 was concentrated and loaded onto Superdex 200 g 16/600 in 20 mM HEPES pH 7.5, 5% glycerol, 150 mM KCl, 2 mM PMSF, and 0.5 mM TCEP. The eluent proteins were concentrated to ~5 mg/ml, snap frozen, and store at −80 °C. Alt-R^®^SpCas9 and Alt-R^®^SpHiFiCas9^25^ are purchased from Integrated DNA Technologies. Alt-R^®^HiFiCas9 contains a single substitution at R691A. Here, we named Alt-R^®^SpCas9 and Alt-R^®^SpHiFiCas9 as ^IDT^SpCas9 and ^IDT^HiFiCas9 in our article. Fluorescent labeling of ^FRET^SpCas9 was performed as described in Sternberg et al. 2015^18^. FRETSpCas9 was mixed with 20-fold excess of Cy3- and Cy5-maleimide (GE Healthcare) dissolved in DMSO, the final concentration of which did not exceed 5%. The reaction was quenched with 10 mM DTT following incubation for 2 h at room temperature and overnight at 4 °C. Labeling reactions were performed in the dark. Labeled ^FRET^SpCas9 was separated via size-exclusion chromatography, concentrated, snap frozen, and stored at −80 °C.

### In vitro sgRNA transcription and refolding

In vitro sgRNA transcription and refolding were performed as described previously^15,31^. The primers used for the templates of sgRNA transcription are listed (Supplementary Table 2). Equimolar concentrations of complementary oligos were mixed in 10 mM Tris-HCl, pH 8.0, 50 mM NaCl, 1 mM EDTA and heated to 95 °C for 5 min, then slowly cooled to room temperature in about 60 minutes. The sgRNA was in vitro transcribed using the HiScribe Qiuk T7 RNA synthesis kit (New England Biolab) following the manufactory protocol. The transcribed sgRNA was further purified using a PureLink column (Thermo Scientific) and refolded by heating to 95 °C and then slowly cooled to room temperature in 10 mM Tris-HCl, pH 8.0, 50 mM NaCl, 1 mM EDTA. We purchased the gRNA for the Singh et al. substrate from Synthego.

### DNA duplex formation and probe labeling

55-nt DNA duplexes were prepared from unmodified DNA oligonucleotides synthesized, and PAGE gel purified by Integrated DNA Technologies. FRET paired-labeled (Cy3/Cy5) DNA oligonucleotides were synthesized, and PAGE gel purified by Gene Link^TM^. A thymine modified with an amine group through a C6 linker (amino dT) was used to label DNA with Cy3 N-hydroxysuccinimido (NHS) or Cy5 NHS. A cytosine modified with an amine group through a C6 linker (amino dC) was used to label DNA with Cy5 NHS. The detailed sequence information is listed in Supplementary Table 2. The tricyclic fluorescent cytidine analog (tC^o^)-labeled DNA oligonucleotides were synthesized, and PAGE gel purified by Bio-Synthesis, Inc. The synthesized oligonucleotides and the position of FRET paired and tC^o^ labeling are listed in the Supplemental Experimental Methods. The DNA duplex used for RuvC cleavage assays was prepared by γ-^32^P labeling or 6-FAM the non-target strand before annealing with the cold complementary strand at a 1:1.15 molar ratio. The DNA duplex used for HNH cleavage assays was prepared by γ-^32^P or 6-FAM labeling the target strand before annealing with cold non-target strand at a 1:1.15 molar ratio.

### Buffer composition for kinetic reactions

Prepared 5X cleavage buffer (100 mM Tris-Cl, pH 7.5, 500 mM KCl, 25% glycerol, 5 mM DTT). After allowing Cas9–gRNA to react with substrate in 1X cleavage buffer (20 mM Tris-Cl, pH 7.5, 100 mM KCl, 5% glycerol, 1 mM DTT) under the conditions for the stopped-flow kinetic, Active-site titration, DNA cleavage and DNA trap cleavage assays.

### Stopped-flow kinetic assay

Stopped-flow experiment was performed as previously described^15,31^. Briefly, Cas9-gRNA complex (1:1 ratio) was mixed with tC^o^-labeled 55/55 nt DNA substrate at 37°C using AutoSF-120 stopped-flow instrument (KinTek Corporation, Austin, TX). The fluorophore was excited at 367 nm, and emission was monitored at 445 nm using a single band-pass filter with 20 nm bandwidth (Semrock) (data in Figs. 2a–f and 3a–f). For the Cy3/Cy5 FRET signal, samples were excited at 550 nm, and the time-dependent fluorescence change was monitored at 670 nm using a single band-pass filter with a 30-nm bandwidth (Semrock) (data in Fig. 3h).

### Global analysis of kinetic data

The kinetic data defining CRISPER-Cas9 cleavage were globally fit using the models shown in our kinetic model (Fig. 5f, inset) by *KinTek Explorer* software (KinTek Corporation, Austin, TX) to obtain rate constants (Figs. 5 and 6, and Supplementary Figs. 10–14). The output for experiments to measure HNH and RuvC DNA cleavage were modeled simply as the sum of products for each cleavage site. For example, for HNH cleavage the products were defined according to our kinetic model (Fig. 5f, inset) as the sum of all species containing P1: EDP1 + EDP1R + EDP1R.Mg +EDP1P2. RuvC cleavage products were defined as the sum of all species containing P2: EDHP2 + EDHP2.Mg+EDP1P2. The fluorescence signal for R-loop formation was modeled as a weighted sum of all species contributing to net fluorescence, with an enhancement in fluorescence in HNH alignment in forming EDH (and subsequent products) and a smaller enhancement in RuvC alignment in forming EDHR (and subsequent products). Specifically, the fluorescence output was modeled as a*(D + ED + b*(EDH + EDP1 + EDH.Mg) +c*(EDHR + EDHR.Mg +EDP1R + EDP1R.Mg +EDHP2 + EDHP2.Mg +EDP1P2)). The scaling factors, *a, b*, and *c* were derived as variable parameters in globally fitting the data. FitSpace confidence contour analysis was performed to define the lower and upper limits for each kinetic parameter and to establish that all parameters, including scaling factors, were well constrained according to this minimal model. This model can be readily expanded to include additional information from new experiments.

### Active-site titration assay

To measure the active-site concentration of SpCas9 in off-target cleavage, a fixed concentration of enzyme (100 nM, estimated from absorbance at 280 nm) of SpCas9.gRNA was allowed to react with various concentrations of off-target DNA (5’ end labeled on the target strand) in the presence of 10 mM Mg^2+^. According to a previous study^9^ and our current results, the intrinsic cleavage rates for SpCas9 off-target cleavage, HypaCas9 and Cas9-HF1 on-target cleavage are slower than SpCas9 on-target cleavage. After 1 hour at 37°C, the products were quenched and resolved on a sequencing gel, quantified, and plotted as a function of DNA concentration (Supplementary Fig. 1b–d). The results showed an active enzyme concentration of 31 nM. Because we used a new preparation of Cas9 enzyme, we also measured the active-site concentration of SpCas9 in on-target cleavage (Supplementary Fig. 1a). The results of IDT Alt-R^®^SpCas9 and Alt-R^®^SpHiFiCas9 are shown in Supplementary Fig. 1e, f).

### DNA cleavage kinetics

To better understand the reaction on the timescale of a single turnover, it is important to design experiments to accurately measure the rate of catalysis. For these measurements, we employed two complementary approaches. In the first case, we examined the time courses of Cas9s on- and off-target cleavage by simultaneously mixing Cas9.gRNA (28 nM active-site concentration) with 10 nM radiolabeled DNA target in the presence of Mg^2+^. After quenching by adding EDTA, we resolved the products on a sequencing gel and quantified the products using a phosphor imager (data in Fig. 1, Supplementary Figs. 2, 3, 5a and 7a, b, e, f, i, j, m, and n). In some experiments we used 6-FAM-labeled DNA and resolved and quantified products using an Applied Biosystems DNA sequencer (ABI 3130xl). By using the first case method, we also can directly measure Cy3/Cy5-labeled DNA cleavage to detect Cy3 signal for target strand (HNH) cleavage from Applied Biosystems DNA sequencer (data in Supplementary Figs. 5b, c and 6). In the second case, we first allow the formation of the Cas9.gRNA.DNA (28 nM active-site concentration with 10 nM radiolabeled or 6-FAM label DNA target) in the absence of Mg^2+^, and then directly measure the cleavage after the addition of Mg^2+^ (data in Supplementary Fig. 7c, d, g, h, k, l, o, p–r). All the detailed protocol information can be found in Liu et al. as described previously^31^. Control experiments using radiolabeled DNA established that the 6-FAM label did not alter the kinetics.

### DNA trap cleavage kinetics

To measure the forward commitment for kinetic partitioning, we employed perfectly matched on-target cold DNA serving as DNA trap. Firstly, Cas9.gRNA (28 nM active-site concentration) and labeled DNA target (10 nM) were incubated in the absence of Mg^2+^ and the reaction was initiated by simultaneously adding Mg^2+^ in the presence or absence of an excess of unlabeled DNA trap (200 nM), and quenched each reaction by the addition of 0.5 M EDTA (data in Fig. 4 and Supplementary Fig. 9).

### Reporting summary

Further information on research design is available in the Nature Research Reporting Summary linked to this article.

## Supplementary information

Supplementary Information

Reporting Summary

Peer Review File

## Data Availability

The data that support the findings of this study are available from the corresponding authors upon request.
