# Supplementary material for: Engineered CRISPR/Cas9 enzymes improve discrimination by slowing DNA cleavage to allow release of off-target DNA
Source: Nat Commun. 2020 Jul 17;11:3576. doi: 10.1038/s41467-020-17411-1 (PMC7367838; doi:10.1038/s41467-020-17411-1)
Supplement: Supplementary file 1 — Supplementary Information [file 41467_2020_17411_MOESM1_ESM.pdf]

Supplementary Information

Engineered CRISPR/Cas9 enzymes improve discrimination by DNA cleavage to allow release of off-target DNA

Liu et al.

Supplementary Figure 1

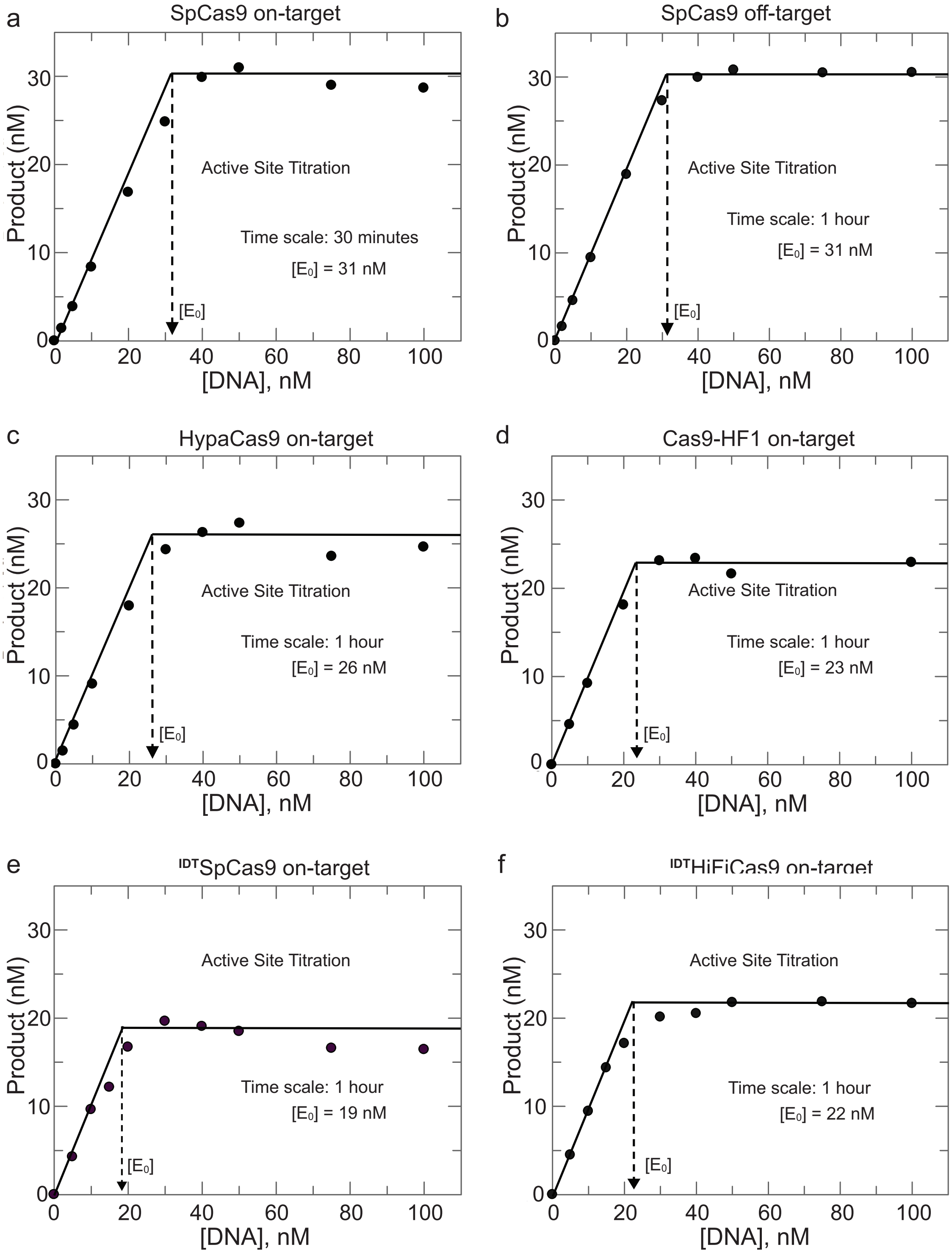

Supplementary Figure 1 | Active-site titration assays for SpCas9, HypaCas9, Cas9-HF1, ^{IDT}SpCas9, and ^{IDT}HiFiCas9. a, An active-site titration was performed by mixing a nominal concentration of 100 nM SpCas9.gRNA (1:1 molar ratio), with variable concentrations of γ-^{32}P-

labeled on-target DNA (0-100 nM). After incubating enzyme with DNA for 10 min at room temperature to form the DNA-binding competent state in the absence of Mg^{2+}, we then added Mg^{2+} and allowed the reaction to run to completion (1 hr) at 37°C. The concentration of DNA product was then quantified as described in Methods and plotted as a function of DNA concentration. The two solid lines indicate titration of the essentially irreversible single turnover reaction, and the vertical dashed arrow shows the active-site concentration of SpCas9.gRNA (31 nM). b, An active-site titration was performed as in (a) but using a nominal concentration of 100 nM SpCas9.gRNA (1:1 molar ratio) with variable concentration of γ-^{32}P-labeled DNA with 3 nt PAM-distal mismatches (off-target) (0-100nM) to give an active site concentration of 31 nM. c, An active-site titration was performed as in (a) but using a nominal concentration of 100 nM HypaCas9.gRNA (1:1 molar ratio) with variable concentration of γ-^{32}P-labeled on-target DNA target (0-100nM) to give an active site concentration of 26 nM. d, An active-site titration was performed as in (a) but using a nominal concentration of 100 nM Cas9-HF1.gRNA (1:1 molar ratio) with variable concentration of 6-FAM-labeled on-target DNA (0-100nM) to give an active site concentration of 23 nM. e, An active-site titration was performed as in (a) but using a nominal concentration of 100 nM ^{IDT}SpCas9.gRNA (1:1 molar ratio) with variable concentration of 6-FAM-labeled on-target DNA (0-100nM) to give an active site concentration of 19 nM. f, An active-site titration was performed as in (a) but using a nominal concentration of 100 nM ^{IDT}HiFiCas9.gRNA (1:1 molar ratio) with variable concentration of a 6-FAM-labeled on-target DNA (0-100nM) to give an active site concentration of 22 nM.

Each titration curve was fit using the quadratic equation:

$$Y = \frac{(E_0 + K_d + DNA_0) \pm \sqrt{(E_0 + K_d + DNA_0)^2 - 4E_0 DNA_0}}{2}$$

where Y represents concentration of cleavage product at the reaction endpoint, E_0 represents the active-site concentration of Cas9, and DNA_0 represents the total concentration of DNA. These data do not define a K_d value because the DNA cleavage is an irreversible reaction that was allowed to proceed to completion. In fitting the data, a nominal K_d value of 0.001 nM was used to generate a fit with essentially irreversible binding and reaction.

Supplementary Figure 2

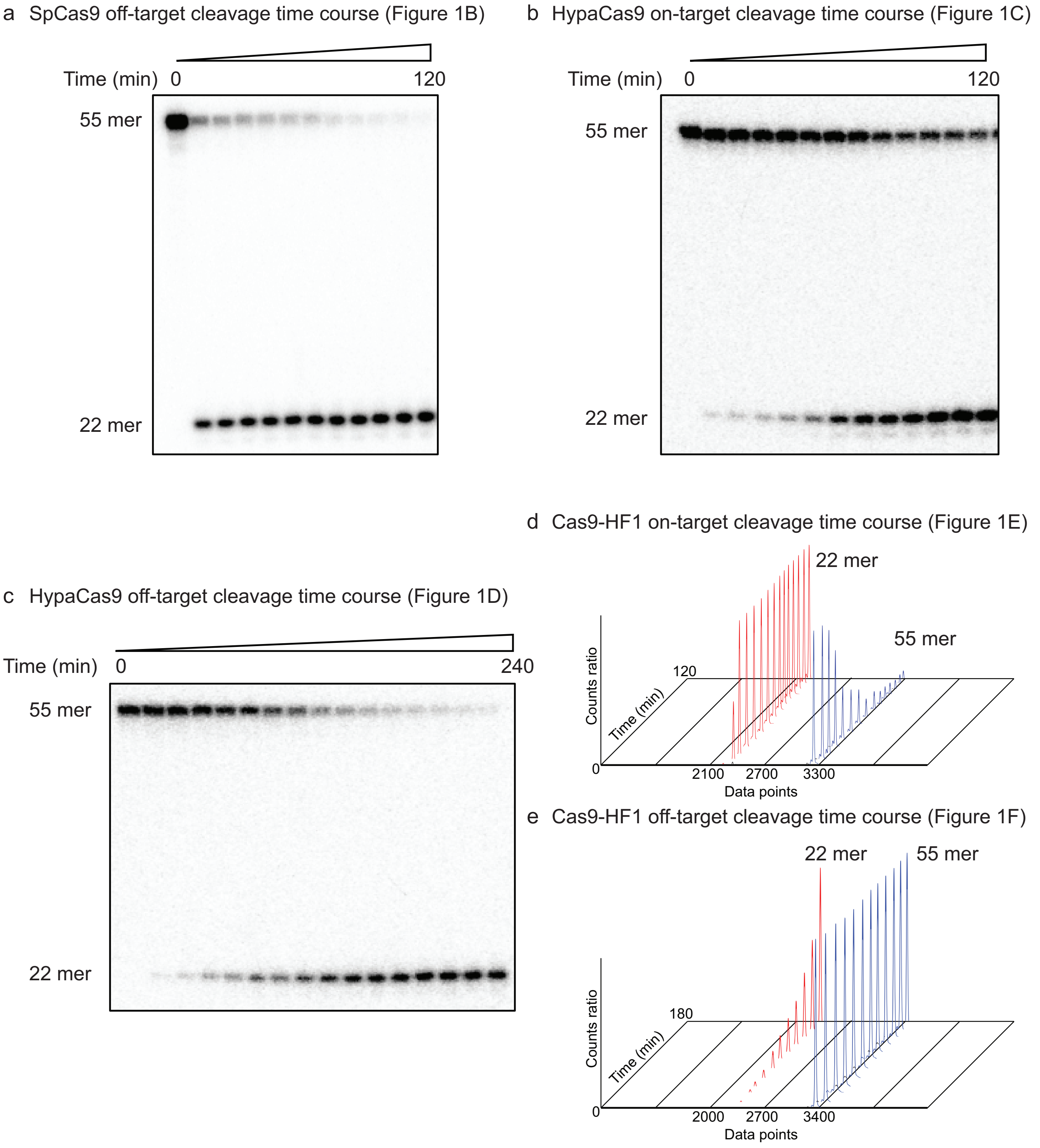

Supplementary Figure 2 | Time-course cleavage assays for SpCas9, HypaCas9, and Cas9-HF1. a–c, Gel images for the data plotted in Fig. 1b (a), 1c (b), and 1d (c), respectively. d–e, Fragment analysis of capillary electrophoresis counts for experiments using 6-FAM-labeled DNA in Fig. 1e (d), and 1f (e). Experiments were repeated independently three times with similar results.

Supplementary Figure 3

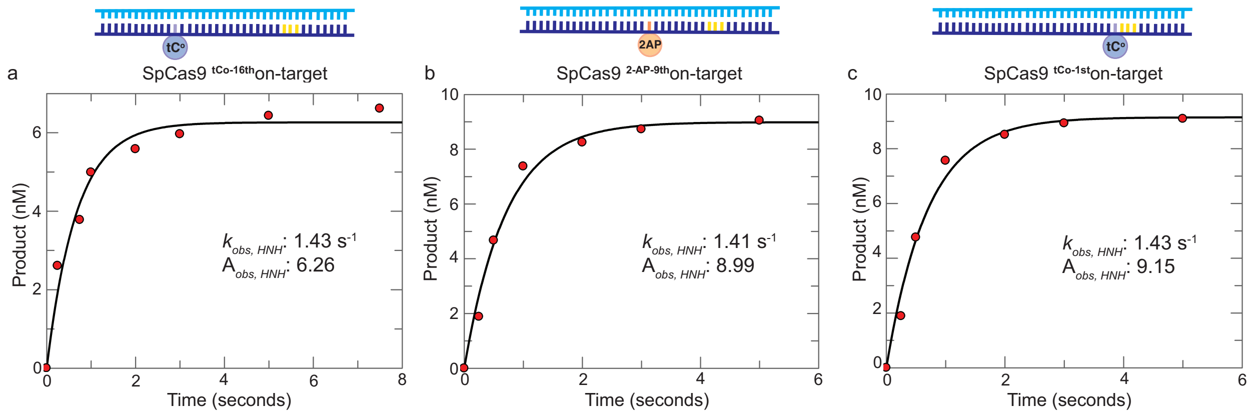

Supplementary Figure 3 | Cleavage assays of target DNA substrates labeled at different positions. a-c, SpCas9-catalyzed DNA cleavage by the HNH domain was monitored after simultaneous addition of on-target DNA and Mg^{2+}. The target strand was labeled with 6-FAM on the 5'-end and the non-target strand contains either the tC° or 2-AP modification at different positions. The kinetics of cleavage are shown for each DNA, labeled at different positions as illustrated by the diagram at the top of each figure. (a) tC° at position -16nt, (b) 2-AP at -9, and (c) tC° at -1 position.

Supplementary Figure 4

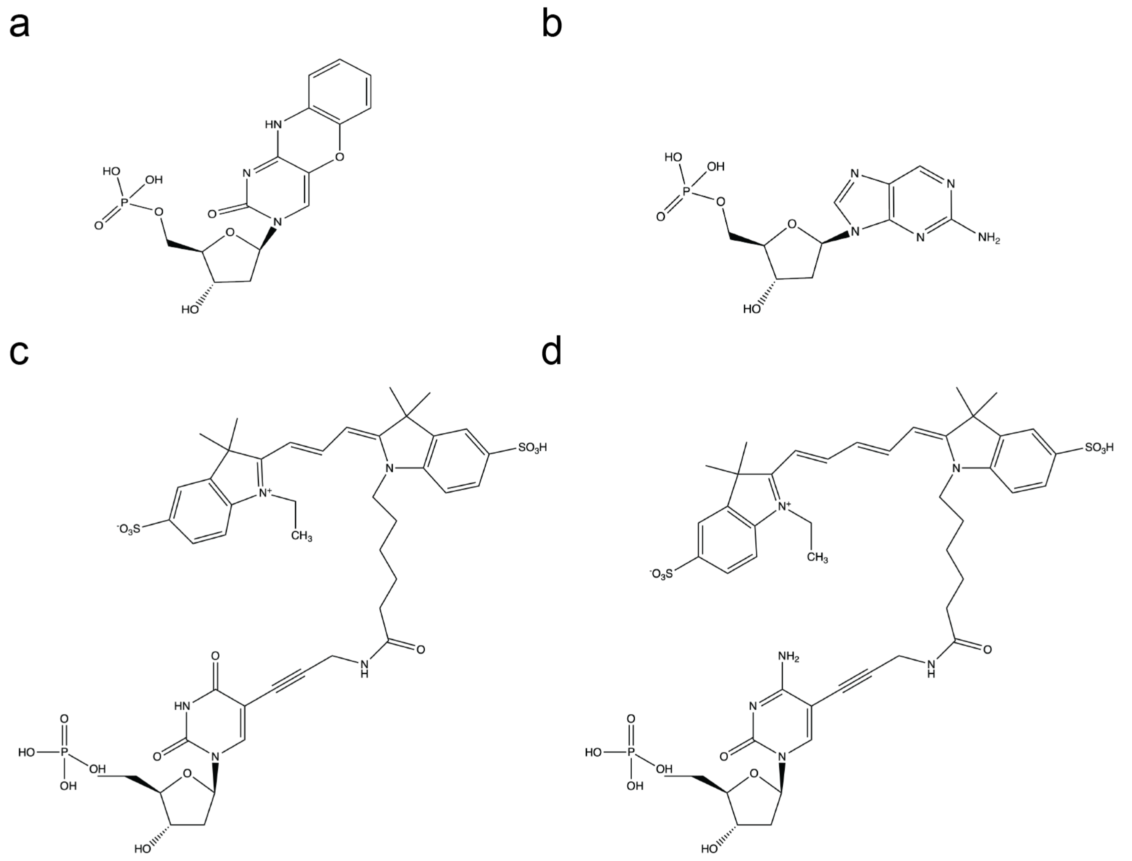

Supplementary Figure 4 | Chemical structures of DNA modified analogue, (a) tricyclic cytosine analogue 1,3-diaza-2-oxophenoxazine (tC^{o}), (b) 2-aminopurine (2AP), (c) amino dT labeled with Cy3 NHS ester, (d) amino dC labeled with Cy5 NHS ester.

Supplementary Figure 5

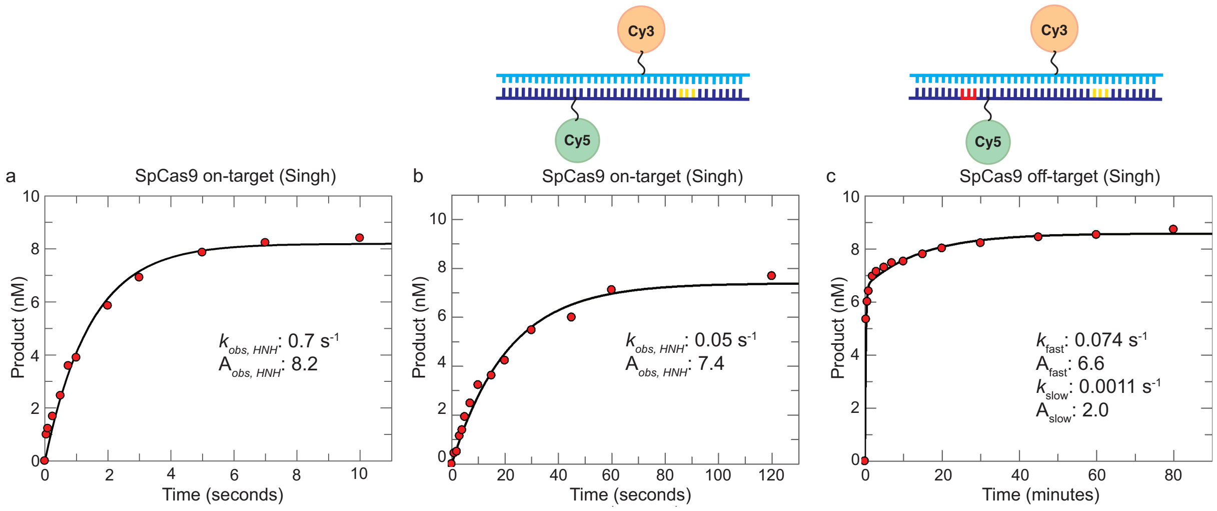

Supplementary Figure 5 l Cleavage assays of Singh *et al*. DNA substrates. a-c, SpCas9-catalyzed DNA cleavage by the HNH domain was monitored after simultaneous addition of on-target DNA and Mg^{2+}. The kinetics of cleavage are shown for each DNA, (a) the target strand was labeled with 6-FAM on the 5'-end, (b) Cy3/Cy5-labeled DNA on-target and (c) off-target substrates.

Supplementary Figure 6

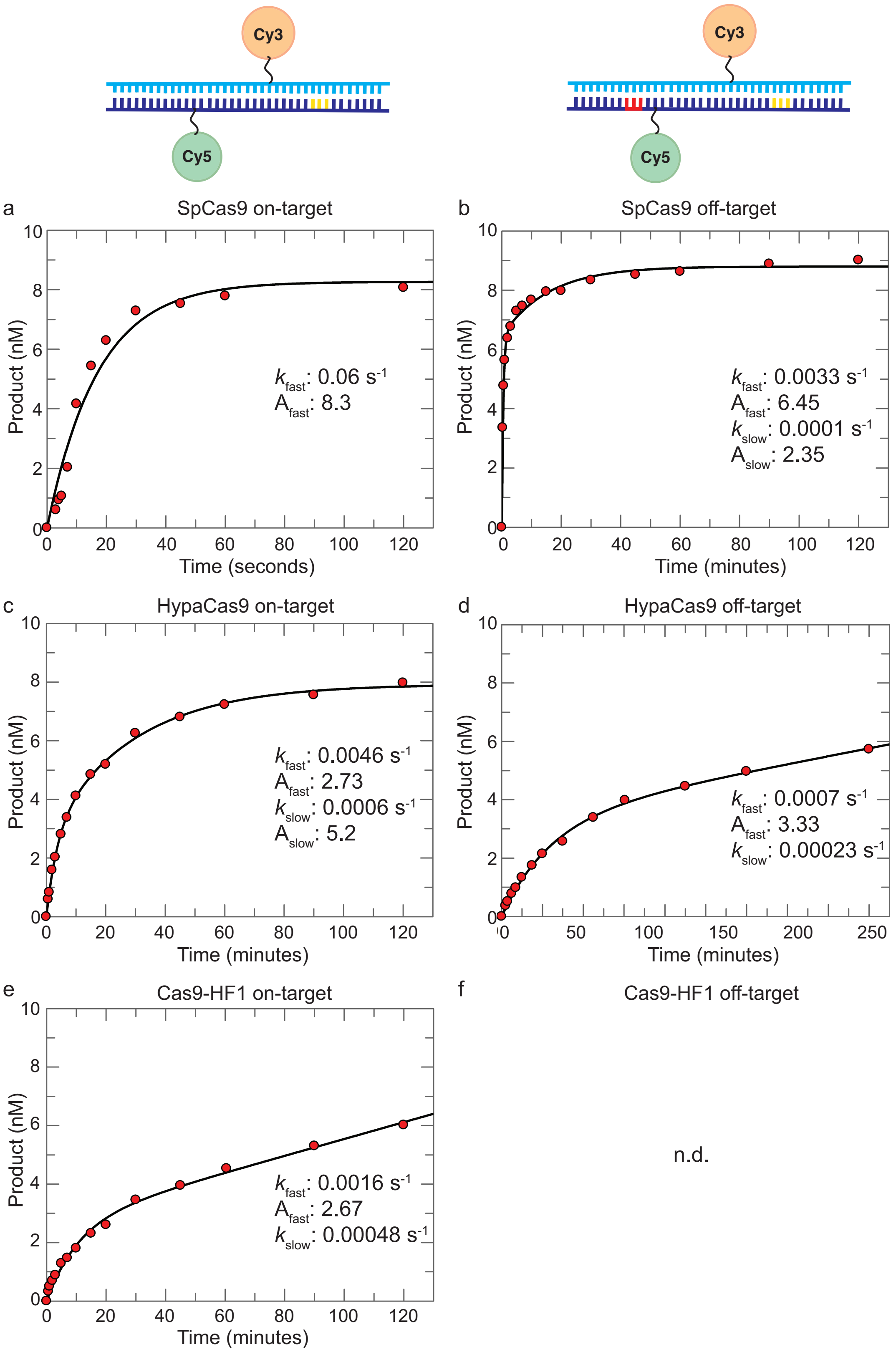

Supplementary Figure 6 | Labeling DNA with a FRET pair decreases the rate of cleavage and increases the fraction of Cas9 in the non-productive state. a–b, Time courses of DNA cleavage catalyzed by SpCas9 were monitored by directly mixing Cy3/Cy5-labeled DNA (10 nM) with SpCas9.gRNA (28 nM active-site concentration) in the presence of 10 mM Mg^{2+} for both on-target (a) and off-target (b) substrates, respectively. Samples were then collected at different time points after stopping the reaction with the addition of EDTA. The concentration of product formed as a function of time was fit to a single-exponential equation. c–d, Time courses of DNA cleavage catalyzed by HypaCas9 were monitored after directly mixing Cy3/Cy5-labeled DNA (10 nM) and HypaCas9.gRNA (28 nM active-site concentration) in the presence of 10 mM Mg^{2+} for on-target (d) and off-target (d) substrates, respectively. The experiments were performed and analyzed as in (a). e–f, Time courses of DNA cleavage by Cas9-HF1 were measured after directly mixing Cy3/Cy5-labeled DNA (10 nM) with Cas9-HF1.gRNA (28 nM active-site concentration) in the presence of 10 mM Mg^{2+} for on-target (e) and off-target (f) substrates, respectively. The Cas9HF-1 off-target cleavage for the FRET paired labeling is not detectable (n.d.), as the chemistry is too slow to detect activity within 3 hours. The experiments were performed and analyzed as in (a).

Supplementary Figure 7

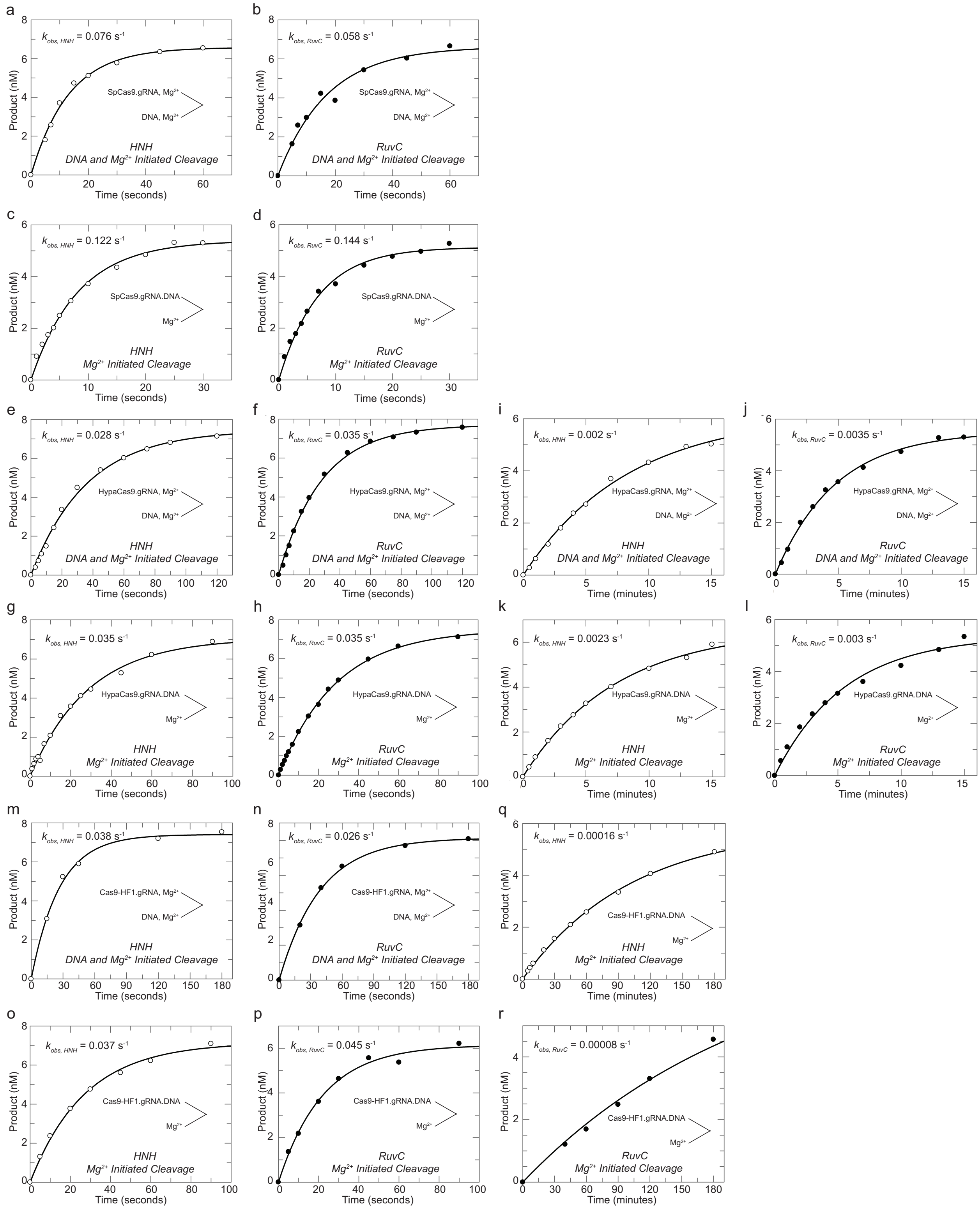

Supplementary Figure 7 | Cleavage rate measurements for SpCas9, HypaCas9, and Cas9-HF1. a–b, SpCas9-catalyzed DNA cleavage at HNH (a) and RuvC (b) domains was monitored

after simultaneous addition of off-target DNA and Mg^{2+}. c–d, SpCas9-catalized DNA cleavage by the HNH (c) and RuvC domains (d) was monitored after pre-equilibration of enzyme with off-target DNA followed by addition of Mg^{2+} to initiate DNA cleavage. e–f, HypaCas9-catalyzed DNA cleavage by the HNH (e) and RuvC (f) domains was monitored after simultaneous addition of on-target DNA and Mg^{2+}. g–h, HypaCas9-catalyzed DNA cleavage by the HNH (g) and RuvC domains (h) was monitored after pre-equilibration of enzyme with on-target DNA and DNA cleavage was initiated by adding Mg^{2}. i–j, HypaCas9-catalyzed DNA cleavage by the HNH (i) and RuvC (j) domains was monitored after simultaneous addition of off-target DNA and Mg^{2+}. k–l, HypaCas9-catayzed DNA cleavage by the HNH (k) and RuvC domains (l) was monitored after pre-equilibration with off-target DNA and then cleavage was initiated by adding Mg^{2+}. m–n, Cas9-HF1-catalyzed DNA cleavage by the HNH (m) and RuvC (n) domains was monitored after simultaneous addition of on-target DNA and Mg^{2+}. o–p, Cas9-HF1-catalyzed DNA cleavage by the HNH (o) and RuvC domains (p) was monitored after pre-equilibration with on-target DNA and then Mg^{2+} was added to initiate cleavage. q–r, Cas9-HF1 cleavage by the HNH (q) and RuvC domains (r) was monitored after pre-equilibration of enzyme with off-target DNA and then Mg^{2+} was added to initiate cleavage.

Supplementary Figure 8

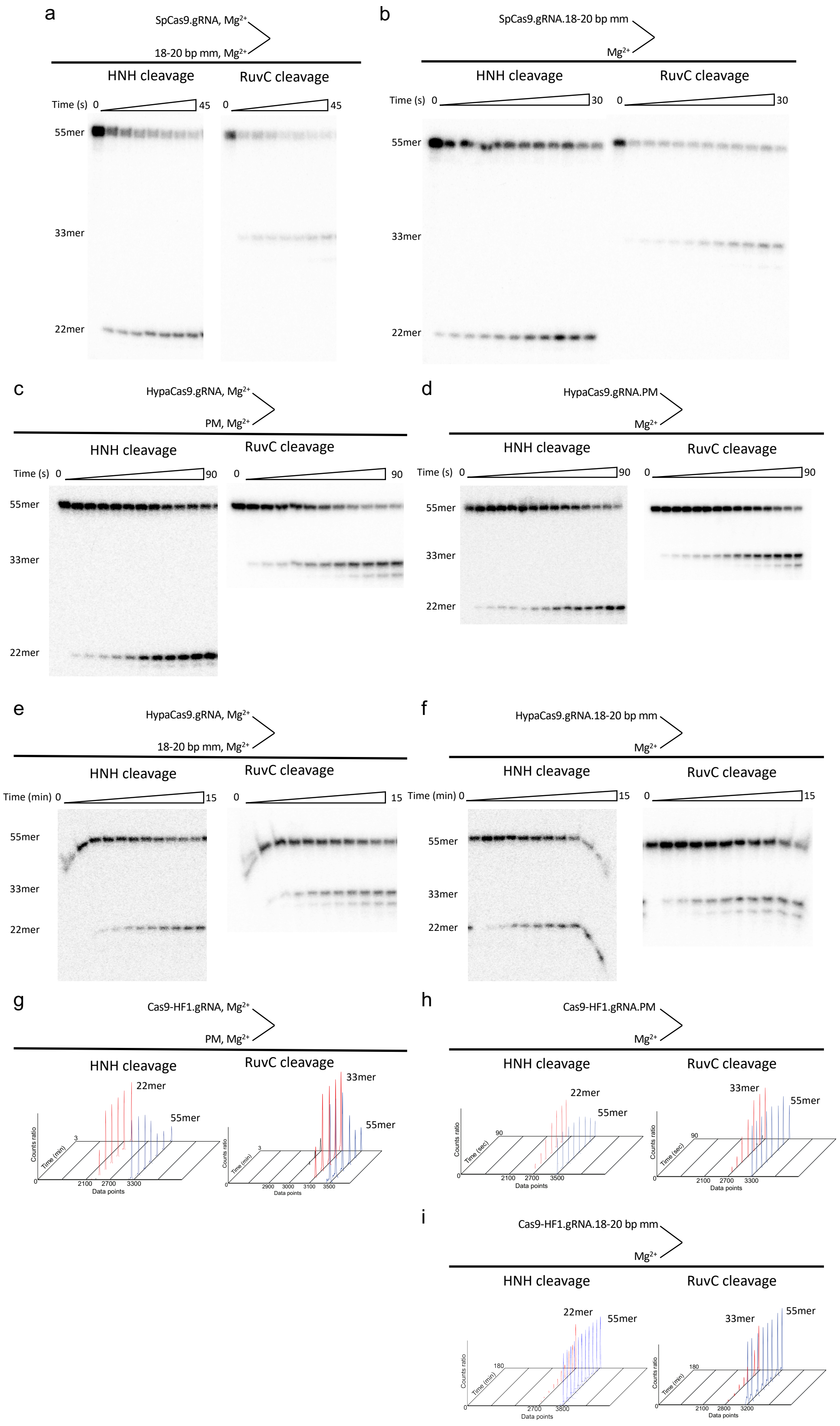

Supplementary Figure 8 | Gel images for data in Supplementary Figure 7. a–b, Gel images for the data plotted in Figs S7a, S7b, S7c, and S7d, respectively. c–d, Gel images for the data plotted in the Figures S7e, S7f, S7g, and S7h, respectively. e–f, Gel images for the data plotted in Figs. S7i, S7j, S7k, and S7l, respectively. g–h, Fragment analysis of capillary electrophoresis counts for experiments using 6-FAM-labeled DNA (HNH) or Atto590-labeled DNA (RuvC) plotted in Figs. S7m, S7n, S7o, and S7p, respectively. i, Fragment analysis of capillary electrophoresis counts for experiments using 6-FAM-labeled DNA (HNH) or Atto590-labeled DNA (RuvC) plotted in Figs. S7q, and S7r, respectively. Experiments were repeated independently three times with similar results.

Supplementary Figure 9

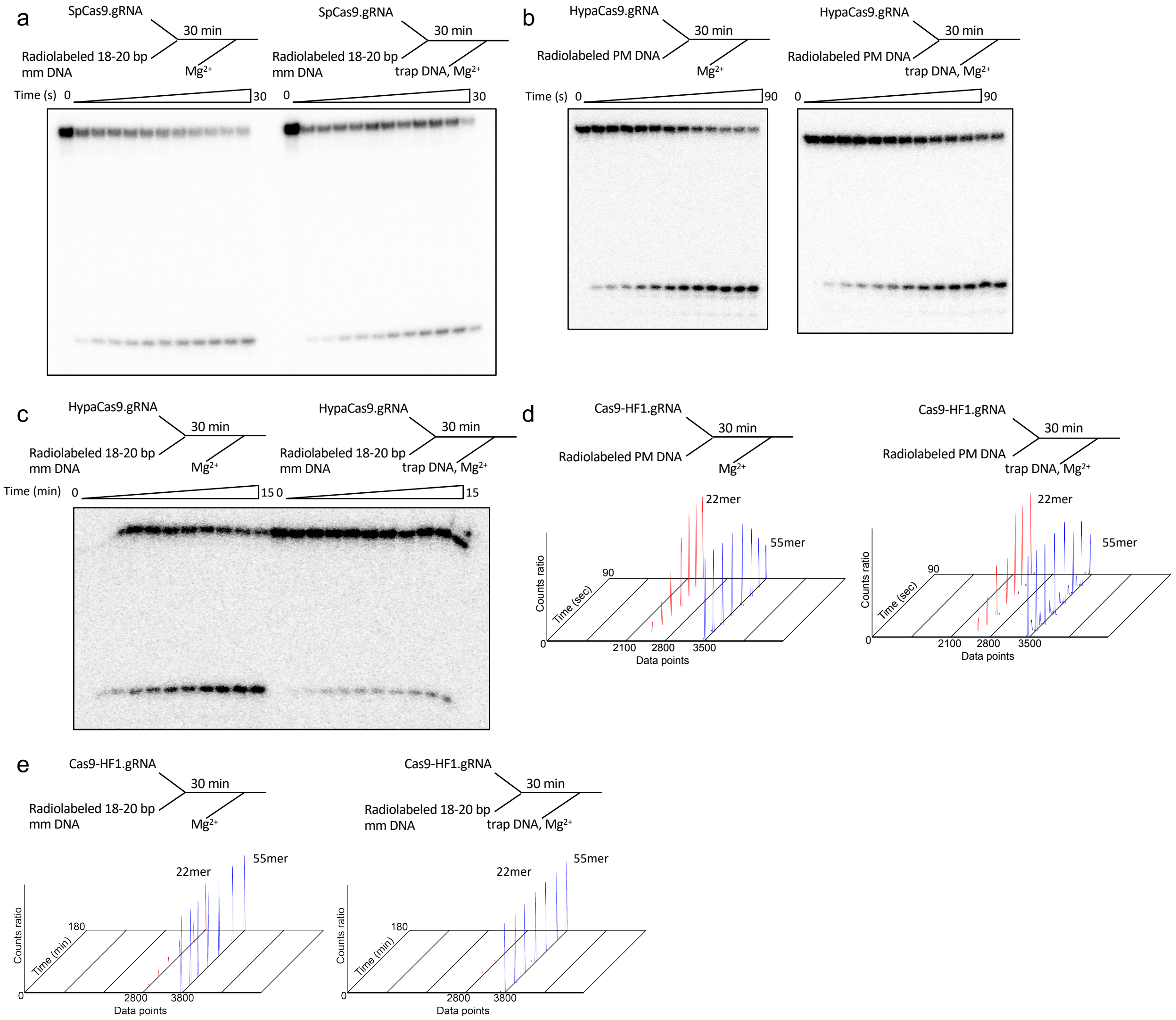

Supplementary Figure 9 | DNA trap assays for SpCas9, HypaCas9, and Cas9-HF1 cleavage. a–c, Gel images for the data plotted in Fig. 4c (a), 4d (b), and 4e (c), respectively. d–e, Fragment analysis of capillary electrophoresis counts for experiments using 6-FAM-labeled DNA in Fig. 4f (d), and 4g (e).

Supplementary Figure 10

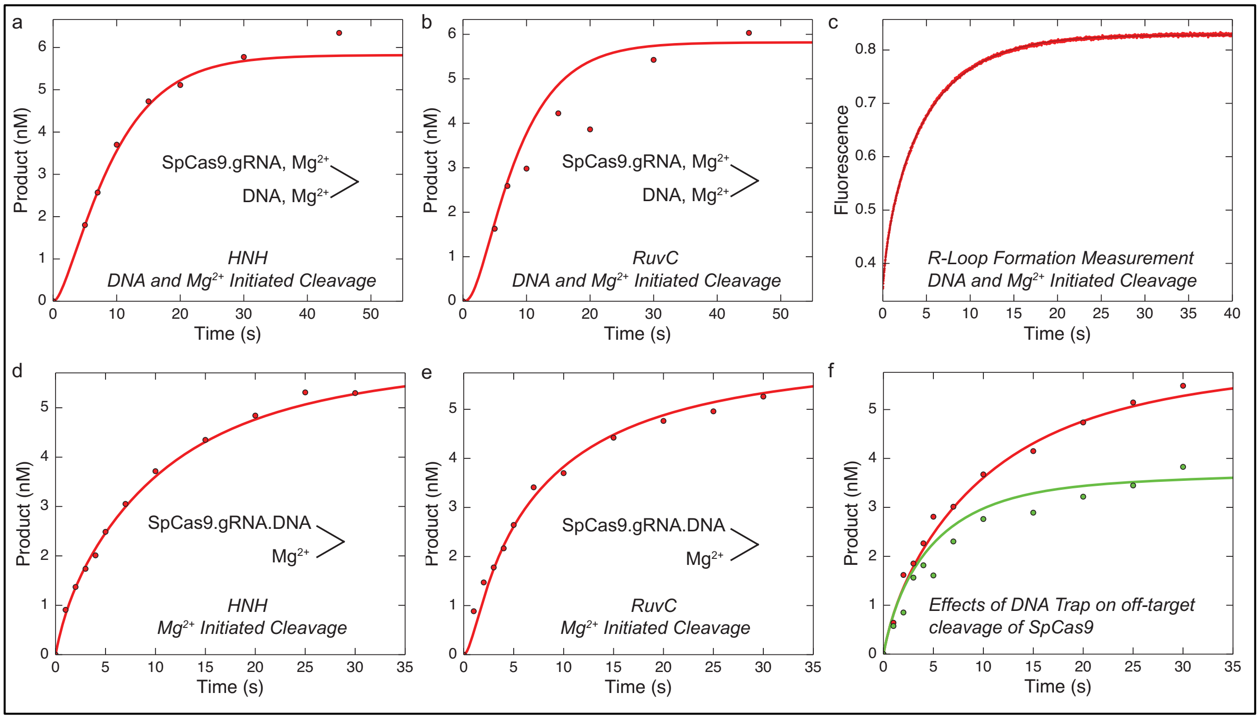

Supplementary Figure 10 | Global fitting of all experiments performed to interrogate off-target activity of SpCas9. The diagrams within each figure show the order of mixing to initiate the reaction. (a) DNA and Mg^{2+} initiated cleavage by the HNH domain. (b) DNA and Mg^{2+} initiated cleavage by the RuvC domain. (c) R-loop formation decay rate measured by tC° fluorescence. d) Mg^{2+} initiated cleavage by the HNH domain. (e) Mg^{2+} initiated cleavage by the RuvC domain. (f) Effect of DNA trap on kinetic partitioning of Cas9 cleavage. The smooth lines were calculated based on the global fit to the data according to Scheme 1 with rate constants shown in Table 1. This global data fitting provides intrinsic rate constants and explains the observed biphasic fluorescence data.

Supplementary Figure 11

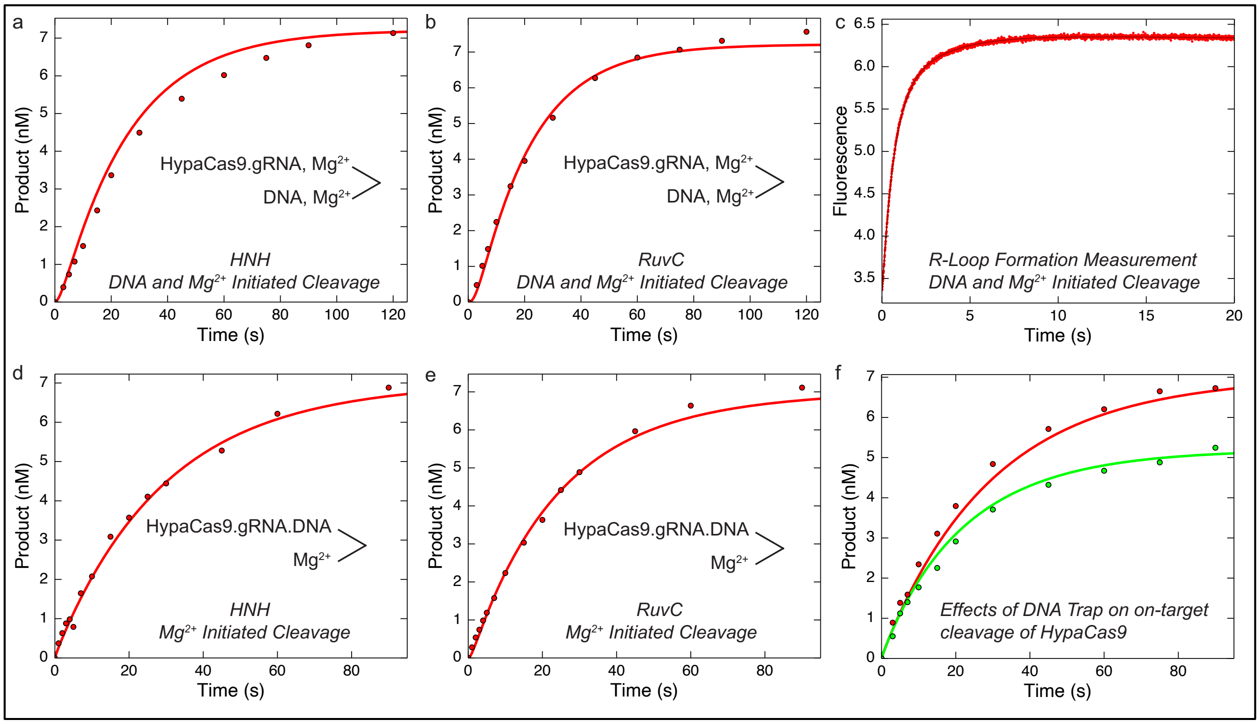

Supplementary Figure 11 | Global fitting of all experiments performed to interrogate on-target activity of HypaCas9. The diagrams within each figure show the order of mixing to initiate the reaction. (a) DNA and Mg^{2+} initiated cleavage by the HNH domain. (b) DNA and Mg^{2+} initiated cleavage by the RuvC domain. (c) R-loop formation decay rate measured by tC^{o} fluorescence. (d) Mg^{2+} initiated cleavage by the HNH domain. (e) Mg^{2+} initiated cleavage by the RuvC domain. (f) Effect of DNA trap on kinetic partitioning of Cas9 cleavage. The smooth lines were calculated based on the global fit to the data according to Scheme 1 with rate constants shown in Table 1. This global data fitting provides intrinsic rate constants and explains the observed biphasic data.

Supplementary Figure 12

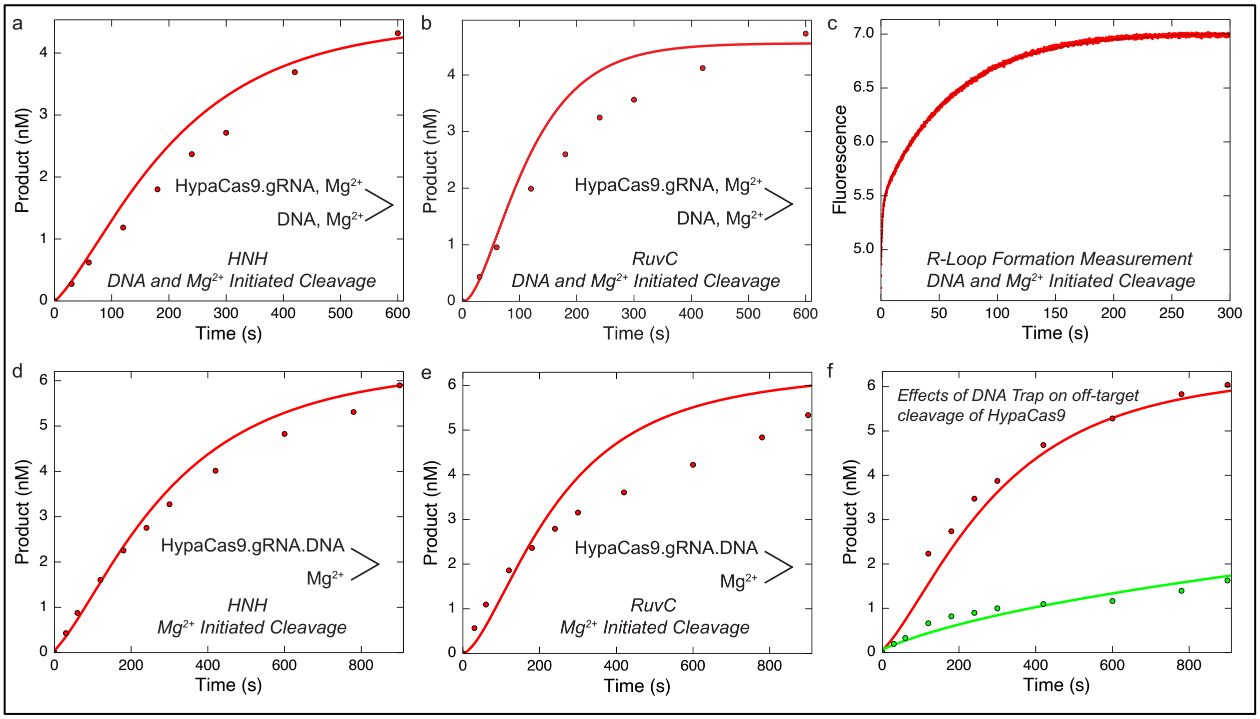

Supplementary Figure 12 | Global fitting of all experiments performed to interrogate off-target activity of HypaCas9. The diagrams within each figure show the order of mixing to initiate the reaction. (a) DNA and Mg^{2+} initiated cleavage by the HNH domain. (b) DNA and Mg^{2+} initiated cleavage by the RuvC domain. (c) R-loop formation decay rate measured by tC° fluorescence. (d) Mg^{2+} initiated cleavage by the HNH domain. (e) Mg^{2+} initiated cleavage by the RuvC domain. (f) Effect of DNA trap on kinetic partitioning of Cas9 cleavage. All curves were calculated based on the global fit to the data according to Scheme 1 with rate constants shown in Table 1. This global data fitting provides intrinsic rate constants and explains the observed biphasic data.

Supplementary Figure 13

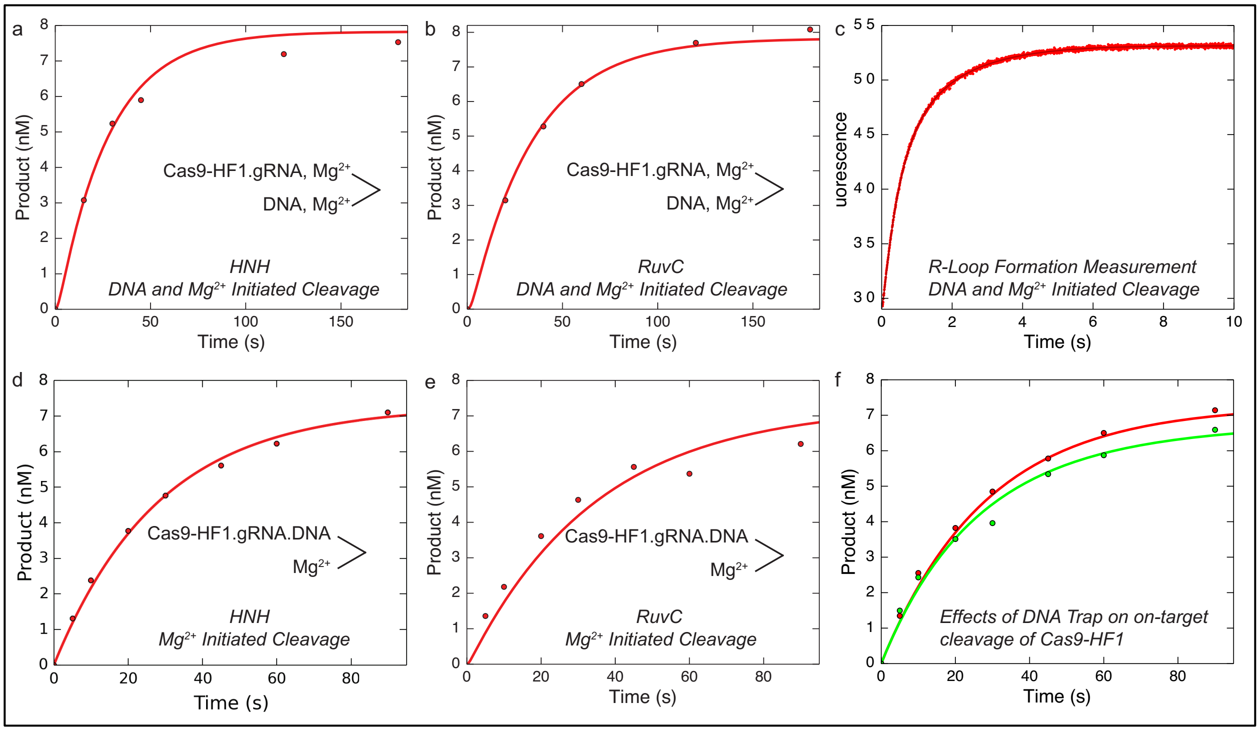

Supplementary Figure 13 | Global fitting of all experiments performed to interrogate on-target activity of Cas9-HF1. The diagrams within each figure show the order of mixing to initiate the reaction. (a) DNA and Mg^{2+} initiated cleavage by the HNH domain. (b) DNA and Mg^{2+} initiated cleavage by the RuvC domain. (c) R-loop formation decay rate measured by tC° fluorescence. (d) Mg^{2+} initiated cleavage by the HNH domain. (e) Mg^{2+} initiated cleavage by the RuvC domain. (f) Effect of DNA trap on kinetic partitioning of Cas9 cleavage. All curves were calculated based on the global fit to the data according to Scheme 1 with rate constants shown in Table 1. This global data fitting provides intrinsic rate constants and explains the observed biphasic data.

Supplementary Figure 14

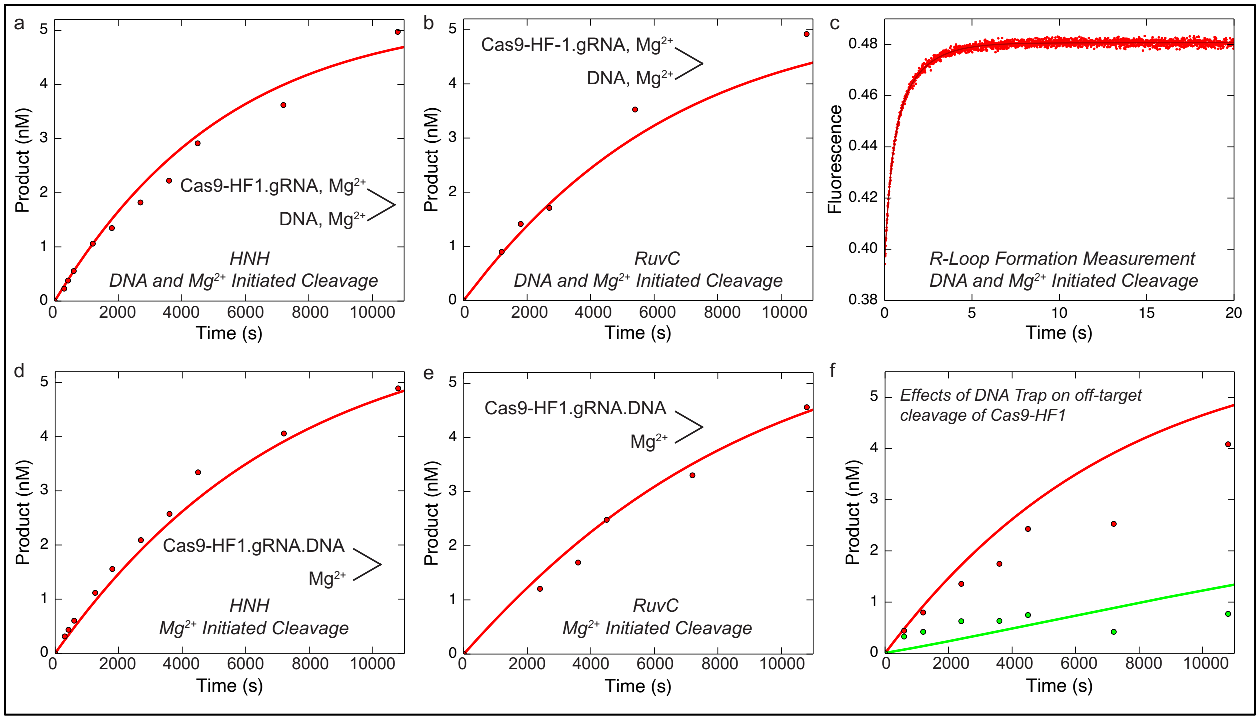

Supplementary Figure 14 | Global fitting of all experiments performed to interrogate off-target activity of Cas9-HF1. The diagrams within each figure show the order of mixing to initiate the reaction. (a) DNA and Mg^{2+} initiated cleavage by the HNH domain. (b) DNA and Mg^{2+} initiated cleavage by the RuvC domain. (c) R-loop formation decay rate measured by tC^{o} fluorescence. (d) Mg^{2+} initiated cleavage by the HNH domain. (e) Mg^{2+} initiated cleavage by the RuvC domain. (f) Effect of DNA trap on kinetic partitioning of Cas9 cleavage. All curves were calculated based on the global fit to the data according to Scheme 1 with rate constants shown in Table 1. This global data fitting provides intrinsic rate constants and explains the observed biphasic data.

Supplementary Table 1 | Calculation of off-rate constants from partitioning (P) and DNA cleavage (k_3).

	On-target			Off-target		
Enzyme	k_3 (s^{-1})	P	$k_{off,calc}$ (s^{-1})	k_3 (s^{-1})	P	$k_{off,calc}$ (s^{-1})
SpCas9	4	0.98	0.098	0.076	0.67	0.037
HypaCas9	0.028	0.75	0.0093	0.0033	0.24	0.011
Cas9-HF1	0.047	0.92	0.0042	0.00016	0.1	0.0014

Supplementary Table 2 | Oligonucleotides used in this study.

Oligonucleotides	Source
DNA target strand (on-target):<br>AGCTGACGTTTGTACTCCAGCGTCTCATCTTTATGCGTCAGCAGAGATTTCTGCT	IDT
DNA non-target strand (on-target):<br>AGCAGAAATCTCTGCTGACGCATAAAGATGAGACGCTGGAGTACAAACGTCAGCT	IDT
DNA target strand (off-target):<br>AGCTGACGTTTGTACTCCAGCGTCTCATCTTTATGCCAGAGCAGAGATTTCTGCT	IDT
DNA non-target strand (off-target):<br>AGCAGAAATCTCTGCTCTGGCATAAAGATGAGACGCTGGAGTACAAACGTCAGCT	IDT
Singh *et al.* DNA target strand (on-target):<br>AGCTGACGTTTGTACTCCAGCGTCTCATCTTTATACATCAGCAGAGATTTCTGCT	IDT
Singh *et al.* DNA non-target strand (on-target):<br>AGCAGAAATCTCTGCTGATGTATAAAGATGAGACGCTGGAGTACAAACGTCAGCT	IDT
Singh *et al.* DNA target strand (off-target):<br>AGCTGACGTTTGTACTCCAGCGTCTCATCTTTATACTAGAGCAGAGATTTCTGCT	IDT
Singh *et al.* DNA non-target strand (off-target):<br>AGCAGAAATCTCTGCTCTAGTATAAAGATGAGACGCTGGAGTACAAACGTCAGCT	IDT
DNA tC$^{\circ}$-labeled non-target strand at -16 nt (on-target):<br>AGCAGAAATCTCTGCTGACGtC$^{\circ}$ATAAAGATGAGACGCTGGAGTACAAACGTCAGCT	Bio-Synthesis, Inc
DNA tC$^{\circ}$-labeled non-target strand at -16 nt (off-target):<br>AGCAGAAATCTCTGCTCTGGtC$^{\circ}$ATAAAGATGAGACGCTGGAGTACAAACGTCAGCT	Bio-Synthesis, Inc
DNA tC$^{\circ}$-labeled non-target strand at -1 nt (on-target):<br>AGCAGAAATCTCTGCTGACGCATAAAGATGAGACGtC$^{\circ}$TGGAGTACAAACGTCAGCT	Bio-Synthesis, Inc
DNA tC$^{\circ}$-labeled non-target strand at -1 nt (off-target):<br>AGCAGAAATCTCTGCTCTGGCATAAAGATGAGACGtC$^{\circ}$TGGAGTACAAACGTCAGCT	Bio-Synthesis, Inc
DNA Cy3-labeled target strand at -6 nt (on-target):<br>AGCTGACGTTTGTACTCCAGCGTCT/Cy3/CATCTTTATGCGTCAGCAGAGATTTCTGCT	Gene Link™
DNA Cy5-labeled non-target strand at -16 nt (on-target):<br>AGCAGAAATCTCTGCTGACGC/Cy5/ATAAAGATGAGACGCTGGAGTACAAACGTCAGCT	Gene Link™
DNA Cy3-labeled target strand at -6 nt (off-target):<br>AGCTGACGTTTGTACTCCAGCGTCT/Cy3/CATCTTTATGCCAGAGCAGAGATTTCTGCT	Gene Link™
DNA Cy-5 labeled non-target strand at -16 nt (off-target):<br>AGCAGAAATCTCTGCTCTGGC/Cy5/ATAAAGATGAGACGCTGGAGTACAAACGTCAGCT	Gene Link™
Singh *et al.* DNA Cy3-labeled target strand at -6 nt (on-target):<br>AGCTGACGTTTGTACTCCAGCGTCT/Cy3/CATCTTTATACATCAGCAGAGATTTCTGCT	Gene Link™
Singh *et al.* DNA Cy5-labeled non-target strand at -16 nt (on-target):<br>AGCAGAAATCTCTGCTGATGT/Cy5/ATAAAGATGAGACGCTGGAGTACAAACGTCAGCT	Gene Link™
Singh *et al.* DNA Cy3-labeled target strand at -6 nt (off-target):<br>AGCTGACGTTTGTACTCCAGCGTCT/Cy3/CATCTTTATACTAGAGCAGAGATTTCTGCT	Gene Link™
Singh *et al.* DNA Cy-5 labeled non-target strand at -16 nt (off-target):<br>AGCAGAAATCTCTGCTCTAGT/Cy5/ATAAAGATGAGACGCTGGAGTACAAACGTCAGCT	Gene Link™
5'-DNA template for sgRNA transcription:<br>AAACAAGCTAATACGACTCACTATAGGACGCATAAAGATGAGACGCGTTTTAGAGCTATGCTGT<br>TTTGGAAACAAAACAGCATAGCAAGTTAAAATAAGGCTAGTCCGTTATCAACTTGAAAAAGTGG<br>CACCGAGTCGGTGCTTTTTTTGGATC	IDT
3'-DNA template for sgRNA transcription:<br>GATCCAAAAAAAGCACCGACTCGGTGCCACTTTTTCAAGTTGATAACGGACTAGCCTTATTTTA<br>ACTTGCTATGCTGTTTTGTTTCCAAAACAGCATAGCTCTAAAACGCGTCTCATCTTTATGCGTC<br>CTATAGTGAGTCGTATTAGCTTGTTT	IDT
Single guide RNA (sgRNA) sequence:<br>GACGCAUAAAGAUGAGACGCGUUUUAGAGCUAUGCUGUUUUGGAAACAAAACAGCAUAGCAAGU<br>UAAAAUAAGGCUAGUCCGUUAUCAACUUGAAAAAGUGGCACCGAGUCGGUGCUUUUUUUGGAUC	IDT
