# Supplementary material for: Engineered CRISPR/Cas9 enzymes improve discrimination by slowing DNA cleavage to allow release of off-target DNA
Source: Nat Commun. 2020 Jul 17;11:3576. doi: 10.1038/s41467-020-17411-1 (PMC7367838; doi:10.1038/s41467-020-17411-1)
Supplement: Supplementary file 3 — Peer Review File [file 41467_2020_17411_MOESM3_ESM.docx]

Peer Review Information

**Journal:** Nature Communications

**Manuscript title:** Click here to enter text.

**Corresponding author name(s):** Click here to enter text.

**Editorial notes:**

**Transferred manuscripts**

This manuscript has been previously reviewed at another journal that is not operating a transparent peer review scheme. This document only contains reviewer comments and rebuttal letters for versions considered at *Nature Communications*.

**Reviewer comments & decisions:**

**Reviewer comments, first version:**

Reviewer #1 (Remarks to the Author):

Review
Engineered CRISPR/Cas9 enzymes improve discrimination by slowing the rate of DNA cleavage to allow release of off-target DNA

In this further revised work, Liu et al. analyzed the target specificity of Streptococcus pyogenes Cas9 (abbreviated here SpCas9, whereas more typical is SpyCas9), a CRISPR enzyme widely used for genome editing and functional screening, together with two recently emerged enhanced-specificity mutants termed high-fidelity Cas9 (Cas9-HF1) and hyper-accurate Cas9 (HypaCas9). Using well-executed ensemble-averaging kinetic approaches, the authors find that DNA unwinding is the rate-limiting step for on-target cleavage by SpCas9, whereas cleavage is 100-fold slower for the high-fidelity mutants so that near-cognate targets often escape cleavage by these variants because they are more likely to partition into dissociation instead. These results differ significantly from a previous publication (Ref. 16; Singh, D. et al. Mechanisms of improved specificity of engineered Cas9s revealed by single-molecule FRET analysis. Nat. Struct. Mol. Biol. 25 (2018) 347), wherein single molecule FRET observations were interpreted as evidence that, at least for the Cas9-HF1 enzyme as well as another engineered high-fidelity variant termed eCas9, DNA rewinding is the rate-determining step responsible for improved off-target discrimination. The current authors explain this discrepancy by a 100-fold decrease in cleavage activity upon introduction of the FRET labels, leading to a critical shift in rate-limiting step that apparently was not controlled for in the 2018 publication.

Major Comments:

This second revision was greatly improved in both representation and message, rendering it almost ready for publication in the opinion of this reviewer, whose recommendations were largely incorporated. In particular, a much more nuanced title, introduction and discussion of the results were achieved. As the authors now acknowledge, “[t]he slower rates of DNA cleavage may be the result of a slower rate of HNH domain movement, an unfavorable equilibrium for the conformational change, and/or a suboptimal alignment of catalytic residues for DNA cleavage”, which was a major point of debate accompanying the previous requests for revision. In addition, the concepts learned are rendered more relevant to the community by explaining that “improvements in Cas9 specificity without sacrificing efficiency may require engineering an enzyme that leads to more rapid release of off-target DNA.” As a whole, the manuscript now appears to fulfill the high expectations for publication in Nature Communications, pending some minor typographical corrections as detailed in the following.

Minor comments:

1. Generally, "cleavage rates" should properly be termed "cleavage rate constants".
2. Page 5 bottom: To measure the intrinsic cleavage rate, we..." (move comma).
3. Page 6, top: "These data indicate that discrimination is based, at least in part, on a change..." (change "by" to "on").
4. Page 7, middle: "Calculation of the apparent dissociation rates (Equation 4) shows that..." (change to "shows").
5. Page 9, top: Typo in "Mg2+".


Reviewer #2 (Remarks to the Author):

The authors have made modest revision of the manuscript. While some of their responses are not fully satisfactory, since their data and interpretation are self-consistent, this reviewer recommends the publication of this work, but only if one remaining concern below is properly addressed by the authors.

This reviewer repetitively asked the authors to describe how they labeled DNA strands for FRET measurements. In this revision, this reviewer still cannot find any description on the labeling procedure other than a blunt description, "The FRET paired-labeled (Cy3/Cy5) DNA oligonucleotides were synthesized, and PAGE gel purified by Gene Link". Without detailed description on the labeling method, readers cannot judge whether the discrepancy between this work and Singh et al is due to the difference in the labeling methods or other factors such as different temperatures. This issue should be resolved before this manuscript is accepted for publication.

**Author rebuttal, first version:**

Reviewer #1 (Remarks to the Author):
Minor comments:

1. Generally, "cleavage rates" should properly be termed "cleavage rate constants"

*We have carefully edited the manuscript to use the term “rate constants” when we are referring to an intrinsic rate constant. In other instances, where we have fit data to an exponential function, we use the term “decay rate” to indicate that the results represent an eigenvalue from data fitting, which is not an intrinsic rate constant. In other instances, we use the term “rate” generically to refer to the speed of the reaction without reference to how it was measured.*

*Each of the following grammatical changes have been addressed:*
2. Page 5 bottom: To measure the intrinsic cleavage rate, we..." (move comma).
3. Page 6, top: "These data indicate that discrimination is based, at least in part, on a change..." (change "by" to "on").
4. Page 7, middle: "Calculation of the apparent dissociation rates (Equation 4) shows that..." (change to "shows").
5. Page 9, top: Typo in "Mg2+".


Reviewer #2 (Remarks to the Author):

The authors have made modest revision of the manuscript. While some of their responses are not fully satisfactory, since their data and interpretation are self-consistent, this reviewer recommends the publication of this work, but only if one remaining concern below is properly addressed by the authors.

This reviewer repetitively asked the authors to describe how they labeled DNA strands for FRET measurements. In this revision, this reviewer still cannot find any description on the labeling procedure other than a blunt description, "The FRET paired-labeled (Cy3/Cy5) DNA oligonucleotides were synthesized, and PAGE gel purified by Gene Link". Without detailed description on the labeling method, readers cannot judge whether the discrepancy between this work and Singh et al is due to the difference in the labeling methods or other factors such as different temperatures. This issue should be resolved before this manuscript is accepted for publication.

*We have added details about how the Cy3/Cy5 DNA was prepared. We thank the reviewer for pointing out this oversight.*

*FRET paired-labeled (Cy3/Cy5) DNA oligonucleotides were synthesized, and PAGE gel purified by Gene Link^TM^. A thymine modified with an amine group through a C6 linker (amino dT) was used to label DNA with Cy3 NHS or Cy5 NHS. A cytosine modified with an amine group through a C6 linker (amino dC) was used to label DNA with Cy5 NHS.*

**Reviewer comments, second version:**

**Author rebuttal, second version:**

**Reviewer comments, third version:**

**Author rebuttal, third version:**
